# The effect of very preterm birth on the Five-Factor Model of personality traits: A meta-analysis of individual participant data

**DOI:** 10.1177/08902070241280101

**Published:** 2024-10-24

**Authors:** Yiwen Liu, Anu Realo, Marina Mendonça, Nicole Baumann, Peter Bartmann, Katri Räikkönen, Kati Heinonen, Rachel Robinson, Neil Marlow, Samantha Johnson, Yanyan Ni, Eero Kajantie, Petteri Hovi, Marjaana Tikanmäki, Dieter Wolke

**Affiliations:** 1 Department of Psychology, 2707 University of Warwick, Coventry, UK; 2 Institute of Psychology, University of Tartu, Tartu, Estonia; 3 Department of Neuroscience, Psychology and Behaviour, College of Life Sciences, 4488 University of Leicester, Leicester, UK; 4 Department of Population Health Sciences, George Davies Centre, 4488 University of Leicester, Leicester, UK; 5 Turner Institute for Brain and Mental Health, School of Psychology Sciences, Monash University, Melbourne, Australia; 6 Department of Neonatology and Pediatric Intensive Care, 39062 University Hospital Bonn, Bonn, Germany; 7 Department of Psychology and Logopedics, 3835 University of Helsinki, Helsinki, Finland; 8 Welfare Sciences/Psychology, Faculty of Social Sciences, Tampere University, Tampere, Finland; 9 UCL Elizabeth Garrett Anderson Institute for Women’s Health, 4919 University College London, London, UK; 10 School of Public Health, LKS Faculty of Medicine, The University of Hong Kong, Hong Kong, China; 11 Department of Chronic Disease Prevention, 3837 Finnish Institute for Health and Welfare, Helsinki, Finland; 12 Clinical Medicine Research Unit, Medical Research Center Oulu, Oulu University Hospital and University of Oulu, Oulu, Finland; 13 Department of Clinical and Molecular Medicine, Faculty of Medicine and Health Sciences, Norwegian University of Science and Technology, Trondheim, Norway; 14 Children’s Hospital, 3835 Helsinki University Hospital and University of Helsinki, Helsinki, Finland; 15 Division of Health Sciences, Warwick Medical School, 2707 University of Warwick, Coventry, UK

**Keywords:** personality, Five-Factor Model, VP/VLBW, individual participant data, neurosensory impairments

## Abstract

There is mixed evidence on personality differences among those born very preterm or with very low birth weight (VP/VLBW). This meta-analysis of individual participant data aimed to examine differences in personality traits between VP/VLBW (*n* = 568) and term-born (*n* = 1,060) adults, and the role of neonatal characteristics and neurosensory impairments in childhood, which have not been previously investigated. Six studies were identified from two research consortia and a systematic search of the literature (PubMed and Scopus); studies were eligible if they included VP/VLBW and term-born adults followed from birth and assessed personality using the Five-Factor Model. Risk of bias (Newcastle-Ottawa Scale) was generally not a concern apart from the use of self-reported measures and the rate of follow-up. Using a one-stage approach, VP/VLBW scored lower on extraversion and openness and higher on neuroticism and agreeableness than term-born participants after adjusting for sex and parental education. Within the VP/VLBW group, those with bronchopulmonary dysplasia scored lower on extraversion and higher on neuroticism, with similar findings after removing participants with neurosensory impairments. Altogether, these findings suggest that a proportion of the effect of VP/VLBW birth on personality may be attributed to neonatal morbidities and altered brain development, although other confounding factors require further research.

## Plain language summary

Adults who were born very prematurely may have different personality compared to adults who were born at full-term. We combined evidence from six different studies to investigate whether these two groups of adults differ in their personality. Furthermore, we also explored the reasons why adults born prematurely may have different personality, such as medical conditions at birth or disabilities in childhood. We primarily identified these studies from a group of research institutes focusing on adults born prematurely, but we also searched the literature for any additional studies that might have been missed. Only studies that recruited both people born prematurely and people who were born at full-term, followed their development into adulthood, and assessed personality across five areas (neuroticism, extraversion, agreeableness, openness, and conscientiousness) were eligible to be included. The studies we included were generally not considered to be biased, apart from the issue that personality was self-reported by the participants, and not all participants who were recruited stayed in the study. We used a statistical technique to combine and analyze data from all studies and found that adults who were born prematurely were less extraverted and open to new experiences, but scored higher on neuroticism and agreeableness, after considering possible sex differences and their parents’ education. Some of these personality differences may be explained by certain medical conditions at birth. In summary, these findings seem to suggest that adults born prematurely may have different personality compared to adults born at full-term, but more research is needed to understand the reasons behind this.

## Introduction

Personality traits can be broadly defined as characteristics of the individual that reflect their consistent patterns of thinking, feelings, and behaving (Funder, 1997; McCrae & Costa, 1987, 2008). The Five-Factor Model is one of the most widely used and comprehensive taxonomies of personality traits that proposes that individual differences in personality can be best captured using five broad traits of neuroticism (e.g., prone to experience negative emotions), extraversion (e.g., being outgoing and sociable), openness to experience (e.g., being imaginative and open to new experiences), agreeableness (e.g., trusting and polite), and conscientiousness (e.g., being thorough and goal oriented) (John et al., 2008).

Over the past decades, several theories have been advanced about how individual differences in personality traits might be associated with biological processes, primarily with the functions of the brain (Cloninger et al., 1993; Depue & Collins, 1999; DeYoung, 2015; Eysenck, 1967; Gray, 1982). However, the majority of these studies relied on neuroimaging studies, which often used relatively small and cross-sectional samples (DeYoung & Blain, 2020). In this paper, we propose another way to investigate predictors of personality traits by using special populations at significant neurobiological risk—those born very preterm or with very low birth weight (VP/VLBW; <32 weeks/<1500 g). We propose to compare personality traits of VP/VLBW with those of contemporary full-term (>37 weeks gestation) control groups to determine how VP/VLBW, with its attendant biological risk and altered development, is associated with differences in personality.

In the following sections, we will briefly provide an overview of the characteristics of VP/VLBW birth, its implication on brain development, and areas of functioning including personality; briefly review the evidence regarding personality differences in the VP/VLBW population; and propose a way of using individual participant data (IPD) from longitudinal birth cohort studies to help understand individual differences in personality.

## VP/VLBW birth and implications for personality development

Around 10.6% of global births are preterm (<37 weeks gestation), and of those, 15% are considered to be very preterm (VP; <32 weeks gestation) or very low birth weight (VLBW; <1,500 g) (Chawanpaiboon et al., 2019). VP/VLBW birth is associated with a number of neonatal complications that may require substantial and prolonged support in intensive care units, and survivors more often suffer a range of brain injuries and alterations in subsequent brain development lasting into adulthood (Bäuml et al., 2015; Costeloe et al., 2012; de Kieviet et al., 2012; Hadaya & Nosarti, 2020; Meng et al., 2016; Volpe, 2009; Wolke et al., 2019). Some of these alterations include white matter injury and reduced gray and white matter in the thalamus and striatum, as well as differences in functional connectivity in cortical and subcortical areas (Bäuml et al., 2015; Meng et al., 2016; Volpe, 2009). A recent meta-analysis contributed further evidence that VP/VLBW adults showed smaller cortical and subcortical volumes compared to term-borns that may explain the lack of catch-up in brain development in adulthood (Kelly et al., 2023). These brain injuries and subsequent alterations in brain development are implicated in a number of functional outcomes, with strong evidence of associations with cognitive and motor performance, and moderate associations with behavioral and emotional problems (Cheong et al., 2020; Eves et al., 2020; Inder et al., 2023; Wolke et al., 2019).

Recent research suggests that alterations in brain development may also have important consequences for personality (DeYoung & Blain, 2020; Dubois et al., 2020). For example, VP/VLBW adults show reduced amygdala volumes in adulthood, which in typically developing adults play a crucial role in the brain’s emotional and stress response system (Schmitz-Koep et al., 2021), and have been associated with higher neuroticism and lower extraversion (Cremers et al., 2011; Holmes et al., 2012; Hu et al., 2020; Kaczkurkin et al., 2016). Openness to experience (openness), characterized by a tendency to seek out new experiences and associated with outcomes such as creativity and mental flexibility, has been associated with larger prefrontal cortex and increased gray matter volume, as well as measures of cognitive function (i.e., IQ) (Abu Raya et al., 2023), all of which are reduced in VP/VLBW adults (Inder et al., 2023; Kelly et al., 2023). Fewer research studies exist on the relationship between altered brain development and agreeableness and conscientiousness. Gray matter volume and white matter integrity have been implicated in some studies (DeYoung & Blain, 2020; Kapogiannis et al., 2013; Riccelli et al., 2017; Xu & Potenza, 2012), but with less certainty compared to other traits, and it remains unclear to what extent brain injury and subsequent alterations in brain development resulting from VP/VLBW birth may relate to these two personality traits.

## Personality differences between VP/VLBW and term-born adults

Given that VP/VLBW birth is associated with significant brain injury and alterations in brain development, which may be implicated in the development of personality, it is plausible that VP/VLBW adults may also show differences on personality traits compared to term-born adults who do not experience the same neurobiological risks. There is some evidence from longitudinal birth cohort studies that VP/VLBW adults scored more highly in neuroticism and agreeableness and lower in extraversion compared to their term-born peers (Allin et al., 2006; Eryigit-Madzwamuse, Strauss et al., 2015; Hertz et al., 2013; Lyall et al., 2016; Schmidt et al., 2008; Waxman et al., 2013) even after accounting for two important predictors of personality differences in the literature, sex, and parental education (Schmitt et al., 2008; Sutin et al., 2017). This is consistent with the direction of effect that would be expected when considering the evidence on the association between brain development and personality (i.e., reduced volume associated with higher neuroticism and lower extraversion). However, these individual studies often had small sample sizes (VP/VLBW being a rare event), and some studies also used different measures of personality (e.g., Eysenck Personality Questionnaire) which did not assess other personality traits such as openness and conscientiousness.

Of the few studies that did examine openness and conscientiousness using the Five-Factor Model, mixed findings have been reported. One study found lower openness and higher conscientiousness in VLBW adults (Pesonen et al., 2008), but this was not replicated in other studies (Eryigit-Madzwamuse, Strauss et al., 2015; Hertz et al., 2013). Lower openness may be expected given its association with cortical volumes and cognitive functioning (Abu Raya et al., 2023), which are both reduced in the VP/VLBW population (Inder et al., 2023; Kelly et al., 2023; Wolke et al., 2019). However, results for conscientiousness require further investigation as there has been less research on the role of brain development in conscientiousness, and therefore weaker evidence for an effect of VP/VLBW birth on this personality trait.

While these studies offer some evidence that VP/VLBW individuals score differently on some personality traits compared to term-born adults, factors that may explain these differences in the VP/VLBW population, such as neonatal characteristics and complications specific to this population, have not been examined. For example, those born VP/VLBW are more likely to have lower birth weight for their gestational age, which may indicate intrauterine growth restriction (Zeitlin et al., 2000), and is associated with further altered brain development compared to those born appropriate for gestational age (Simões et al., 2017). VP/VLBW infants are also at increased risk of neonatal complications such as bronchopulmonary dysplasia—a chronic lung disease that requires supplemental oxygen treatment (Hedderich et al., 2020)—which may contribute to brain injury of the immature brain (Neubauer et al., 2015; Schmidt, 2003; Siffel et al., 2021). Furthermore, VP/VLBW infants are also more likely to have complications which may result in delivery through elective or emergency caesarean section (Zeitlin et al., 2010). In term-born infants, this has been associated with increased neurodevelopmental challenges including delays in motor skills, difficulties in social and communication skills, and autism spectrum disorder (Curran et al., 2015; Al Khalaf et al., 2015), although evidence in VP/VLBW populations remain mixed (Blake et al., 2021). The factors that are associated with altered brain development may thus help explain individual differences in personality scores in the VP/VLBW population. These same factors are also associated with qualitatively more severe neurosensory impairments in childhood, such as cerebral palsy, deafness, blindness, or cognitive impairments (Draper et al., 2020; Eves et al., 2020; Johnson et al., 2009; Marlow et al., 2005; Wolke et al., 2019). To summarize, previous studies in the VP/VLBW population have focused on just describing personality differences between VP/VLBW and term-born adults. To advance what factors may associate with personality traits, it will be important to examine the contributions of neonatal characteristics and complications on personality traits. Furthermore, are personality differences in the VP/VLBW population associated with widely altered brain development rather than severe neurological insult; that is, do differences remain after excluding those with neurosensory impairments?

## Current study

The aim of this study was to utilize the power of individual participant data (IPD) meta-analysis to examine differences in personality traits (outcome of interest) between VP/VLBW (population and exposure) and term-born individuals (comparator). This is part of a collection of IPD meta-analyses which investigated VP/VLBW and other functional outcomes in adulthood (i.e., IQ) using data from the same research consortium (Eves et al., 2021). An IPD meta-analysis overcomes challenges with traditional meta-analysis of aggregate data from published studies and reduces publication bias by including both published and unpublished data (Burke et al., 2017; Debray et al., 2015). Furthermore, an IPD benefits from having increased statistical power to perform subgroup analysis (Eves et al., 2021; Lambert et al., 2002), to examine whether personality differences in the VP/VLBW population may be additionally explained by other risk factors.

The following three research objectives were pursued: first, we examined differences in each personality trait between VP/VLBW and term-born individuals, to investigate whether VP/VLBW adults show differences in personality traits relative to term-born individuals. In line with previous research and the expected direction of effect from the association between altered brain development and personality, we hypothesized that VP/VLBW individuals would score lower on extraversion and higher on neuroticism compared to term-born individuals, but uncertain on the direction of effect for the other personality traits given the mixed findings in the literature. To reduce potential confounding bias, we adjusted for the effects of sex and parental education (as a proxy for socio-economic status), given that both have been shown to be important predictors of personality traits in previous research (Schmitt et al., 2008; Sutin et al., 2017).

Second, we investigated the contribution of neonatal characteristics (i.e., gestational age, birth weight, and delivery mode) and complications (i.e., bronchopulmonary dysplasia)—which may act as proxy measures indicating the severity of possible alterations in brain development—in explaining differences in personality scores within the VP/VLBW population, and hypothesize that lower gestational age and birth weight and caesarean section (instead of vaginal) as well as the presence of bronchopulmonary dysplasia (indicating more severe complications and associated with altered brain development), would be associated with lower scores on extraversion and openness, and higher scores on neuroticism, agreeableness, and conscientiousness.

Lastly, as VP/VLBW birth is associated with impairment and functional disabilities in childhood that may impact personality traits, we repeated the above analyses by excluding VP/VLBW participants with childhood neurosensory impairments to examine to what extent the association between VP/VLBW birth or neonatal characteristics/complications and personality traits remain after excluding those with moderate to severe disabilities.

## Methods

The protocol is registered at the University of York Centre for Reviews and Dissemination (PROSPERO), registration number CRD42020162046 (https://www.crd.york.ac.uk/prospero/display_record.php?ID=CRD42020162046). This study is reported according to the Preferred Reporting Items for Systematic Review and Meta-Analyses (PRISMA) guidelines for individual participant data (Stewart et al., 2015).

### Eligibility criteria and search strategy

Eligibility criteria were considered in relation to the PICO (population, intervention/exposure, comparator, and outcome) framework. Cohorts were eligible for inclusion if they: (1) included VP/VLBW participants (both the population and exposure); (2) included term-born participants in the same study (comparator/control group), and prospectively followed both groups from birth into adulthood (18 years or older); and (3) assessed personality using the Five-Factor Model (outcome). Cohorts were initially identified from the RECAP-preterm (Research on European Children and Adults Born Preterm, https://recap-preterm.eu/) and APIC (Adults Born Preterm International Collaboration, https://www.apic-preterm.org/) consortia, two large research collaborations across Europe, North America, and Australasia, which the authors were also members of. The two consortia included 13 cohort studies; all studies received ethical approval in their respective country and participants provided written informed consent.

We also conducted a literature search to identify other cohorts outside RECAP/APIC that were eligible for inclusion. We searched PubMed using the following keywords: personality[Title/Abstract] AND (preterm[Title/Abstract] OR low birth weight[Title/Abstract]) AND adult*[Title/Abstract]. The search was performed up to October 2020, and an updated search was carried out to November 2023. We further searched Scopus up to November 2023 using the same terms within the article title, abstract and keywords section (personality AND (preterm OR low birth weight) AND adult*).

### Study selection, data extraction, and harmonization

For eligible studies within the RECAP/APIC consortia, data dictionaries for neonatal measures and adult outcomes were obtained from each cohort, and those eligible for inclusion were contacted for involvement in the study. Two authors (YL and MM) examined the data dictionaries to ensure all relevant cohorts were identified.

Two authors (YL and AR) also examined the eligibility of studies identified from the literature search, and any disagreements were resolved via discussion. Relevant studies identified through the literature search were contacted to request individual participant data and combined with the RECAP/APIC studies.

Data were transferred under signed grant agreements and individual data transfer agreements to the University of Warwick. Item-level data were requested where possible.

#### Personality assessment

Personality was the main outcome and assessed across five broad traits within the Five-Factor Model: neuroticism, extraversion, openness, agreeableness, and conscientiousness. All assessments were self-reported by participants in adulthood. Two main personality measures were considered: the Big Five Inventory (BFI) (John et al., 1991) and the NEO Personality Inventory (NEO-PI) (McCrae & Costa, 1985).

The BFI was developed by John et al. (1991) and contained 44 items rated on a 5-point Likert scale, with 1 representing strongly disagree and 5 strongly agree. A short version, the BFI-10, was also developed subsequently and contained two items for each personality trait. These items were selected based on a number of criteria including item-total correlations and factor loadings. The BFI-10 has been validated in both the United States and Germany using the English and German versions (Benet-Martínez & John, 1998; Rammstedt et al., 2013; Rammstedt & John, 2007).

The NEO-PI was developed by Costa and McCrae (1989) and contained 181 items rated on a similar 5-point Likert scale to the BFI. As well as assessing the five broad personality traits, items were also designed to assess 18 specific facets within the five personality domains. The NEO-PI has been translated and validated in multiple languages, such as the NESTA, a 181-item authorized Finnish version (Hämäläinen, 1994). Variations of the NEO-PI also include the revised NEO-PI (NEO-PI-R; 240 items) (Costa & McCrae, 1992), the NEO Five-Factor Inventory (NEO-FFI; 60 items) (Costa & McCrae, 1989), and the revised version of the NEO-FFI (NEO-FFI-R; 60 items), where 14 items from the NEO-PI was replaced by items from the NEO-PI-R (McCrae & Costa, 2004).

The comparability of the BFI and NEO-PI have been examined in the literature: the majority of items on the BFI-10 were found to be moderately correlated with the relevant scales on the NEO-PI-R, with stronger correlation between the two measures on neuroticism, extraversion, and conscientiousness (*r* = .50–.56) and weaker correlation for openness and agreeableness (*r* = .40) (Rammstedt & John, 2007; Schmitt et al., 2007). When the full BFI scale was compared with the NEO-PI-R, stronger correlation was seen with agreeableness (*r* = .76) (Miller et al., 2011), suggesting that the weaker correlation found between the BFI-10 and NEO-PI-R may be due to the loss of information from the reduction of items in this domain (Rammstedt & John, 2007; Schmitt et al., 2007).

Item-level data were extracted from all cohorts included in the study if available, and mean scores were calculated for each of the five personality traits, with higher scores indicating higher levels of neuroticism, extraversion, openness, agreeableness, and conscientiousness. Cronbach’s alpha was used to estimate internal consistency or reliability of each of the five personality traits (if three or more items were used). When only two items were used to assess a personality domain, then Pearson correlation coefficient was calculated instead, and if a weak correlation was found (defined as *r* < .25), then only the item which did not require reverse coding was used to represent scores on that domain, rather than calculating the average. Mean scores were subsequently transformed into *z*-scores using the mean and standard deviation of term-born participants as the reference within each cohort, so that results in the VP/VLBW population can be interpreted as being relative to term-born individuals.

### Main variables used in the analysis

#### Predictors

##### VP/VLBW vs term-born groups

The main predictor of interest was VP/VLBW birth obtained from birth records. VP/VLBW birth was defined as gestational age below 32 completed weeks or birth weight below 1,500 g. Healthy term-born participants were specified by each cohort and had gestational age equal to or greater than 37 weeks.

#### Covariates available for the whole population

##### Sex

Male (as determined at birth) was the reference group. This was included as a covariate in the study as there is evidence to suggest that males and females show different personality profiles (Schmitt et al., 2017).

##### Parental educational level

Classified as low, medium, or high according to the ISCED (International Standard Classification of Education) classification, where low education level is equivalent to ISCED level 0 to 2, medium education level equivalent to ISCED level 3 to 5, and high education level (reference group) equivalent to ISCED level 6 to 8 (UNESCO Institute for Statistics, 2012). This was reported either during participants’ childhood or in adulthood if data was not available in childhood and was used as a proxy measure for parental education level in childhood. Maternal education was used where possible, otherwise parental education (combined maternal and paternal) was used.

#### Predictors specific to VP/VLBW population

##### Gestational age

Gestational age is calculated as completed weeks, with lower age indicating potentially more severe altered brain development.

##### Birth weight for gestational age

Determined using the Fenton reference (Fenton & Kim, 2013), where sex-specific z-scores were calculated from LMS (lambda, Mu, and Sigma) parameters generated using the Fenton growth reference chart (Fenton & Kim, 2013; Fenton & Sauve, 2007)

##### Delivery mode

Mode of delivery was categorized as either vaginal delivery or caesarean section, with vaginal delivery as the reference group.

##### Bronchopulmonary dysplasia

The presence of bronchopulmonary dysplasia was defined as receipt of supplemental oxygen either for more than 28 days after birth or still at 36 weeks postmenstrual age depending on the study.

##### Neurosensory impairments in childhood

This was a composite measure which consisted of having any of the following: any cerebral palsy, visual impairment (blind/any visual disability vs not blind or no visual disability), hearing impairment defined as deafness that could not be corrected by hearing aids, and cognitive disability defined as scoring less than 70 in a standardized IQ (intelligent quotient) test in childhood, or a proxy measure for cognitive disability was used if childhood IQ was not available. This was a dichotomous variable with either no impairments or the presence of any impairment.

### IPD integrity and risk of bias

Data were checked for completeness and any issues such as the coding of items were resolved by communicating with the study investigators. Two authors (YL and MM) used the Newcastle-Ottawa Scale (Wells et al., 2012), which is the recommended Cochrane tool, to assess the quality of each study (Supplementary Materials, Table S1), and any disagreements were resolved through discussions. We had access to all data from cohorts within the RECAP and APIC consortium; therefore, selective outcome reporting was generally not a problem. We further performed a secondary analysis to include aggregate data from the Danish cohort study as individual-level data were not available.

### Statistical analyses

#### Primary analysis (one-stage IPD)

Statistical analyses were carried out using R version 3.6.3. Data were analyzed using a linear mixed model one-stage IPD approach, where all data are analyzed in a single step with added random effects to account for differences between cohorts (Burke et al., 2017). This has been shown to benefit from increased statistical power compared to a two-stage approach, especially when covariates are included in the model (Kontopantelis, 2018), or when studies have small sample sizes (Riley et al., 2023), and allows the detection of individual-level factors (Eves et al., 2021; Tierney et al., 2015). This approach has been previously used in another IPD meta-analysis which investigated VP/VLBW and IQ in adulthood using data from the same research consortium (Eves et al., 2021).

For our first research objective, we examined unadjusted standardized mean differences between VP/VLBW and term-born participants for each trait, using the R package “lme4” (Bates et al., 2015). VP/VLBW birth was entered as a fixed effect, and cohort-level information (year of study, age of assessments, and rates of attrition) were entered as random effects in a random intercept model (nested by cohort-level information). We further adjusted the model for sex and parental education and examined the interaction between VP/VLBW birth and sex/parental education. For our second research objective, we conducted subgroup analyses within the VP/VLBW population to examine the effects of neonatal characteristics (gestational age, birth weight *z-*scores, and delivery mode), neonatal complications (bronchopulmonary dysplasia) on each trait in the VP/VLBW group, adjusted for sex, and parental education. For our third research objective, we repeated the above subgroup analyses after removing VP/VLBW participants who had neurosensory impairments in childhood.

Missing data on any predictor variables were handled using multiple imputation by chained equation (“mice”) in R (Buuren & Groothuis-oudshoorn, 2011), with 40 imputed datasets. Only data on parental education, delivery mode and bronchopulmonary dysplasia were missing; therefore, only these variables were imputed. Results were pooled together using the “pool()” function in R, which combined estimates from all imputed datasets (Buuren & Groothuis-oudshoorn, 2011). Overall, the percentage of missing data imputed was 19.9% (see Table S2 for number of cases imputed for each cohort).

Heterogeneity was assessed in the one-stage IPD using τ_00_ and σ^2^ (variance components), where larger values of τ_00_ indicate larger between-cohort variance, and larger values of σ^2^ indicate larger residual variance (Kreft & de Leeuw, 1998).

#### Secondary analysis (two-stage IPD)

We further performed two-stage meta-analysis by including aggregate data from studies for which raw data were not available. We performed a two-stage meta-analysis using the R package “metafor” and fitted a random effect model using effect sizes from each study. Findings are visualized using forest plots. Heterogeneity was assessed in the two-stage IPD using I^2^, which measures the percentage of variation across different cohorts due to heterogeneity rather than chance (Deeks et al., 2019). This measurement of heterogeneity is different to the one-stage meta-analysis, which estimates the proportion of total variance in the underlying distribution of true effect sizes, whereas the I^2^ measures the percentage of variability that’s not caused by chance alone (Huang, 2023). Interpretation of I^2^ was based on the Cochrane handbook, where 0%–40% suggest little heterogeneity, 30%–60% suggest moderate heterogeneity, and 50%–90% substantial heterogeneity (Deeks et al., 2019).

#### Robustness analysis

To check whether different definitions of bronchopulmonary dysplasia may affect our findings, we further repeated analyses within the VP/VLBW population by removing cohorts which used a different definition (i.e., oxygen supply at 36 weeks postmenstrual age) compared to others (i.e., supply of oxygen for more than 28 days after birth).

We also conducted a two-stage IPD using only cohorts included in the one-stage IPD, to check whether findings remained similar using different methodologies.

### Deviations from pre-registration

Risk-taking and delinquent behaviors were originally proposed to be secondary outcomes. However, delays in data transfer agreements led to a lack of time available within the funding period to complete the project; therefore, these secondary outcomes were not investigated.

We originally proposed to investigate sex differences in personality as a separate research question. However, sex was included as a covariate instead of a predictor, which is in line with our original aim of accounting for the role of sex, but instead focuses on whether differences in personality between VP/VLBW and term-born adults remain after controlling for the effect of sex (rather than examining sex differences in personality per se). We additionally included parental education as a covariate given its role in personality in the literature.

Lastly, we excluded VP/VLBW participants with neurosensory impairments in childhood, which differed from our original hypothesis of investigating the moderating role of neurosensory impairments. Neurosensory impairments in childhood were a composite measure consisting of multiple indicators, which makes interpretation difficult given the heterogeneous nature of the group. Therefore, rather than including it as a variable in the analyses, we instead repeated our analyses by removing participants with neurosensory impairments, to check whether personality differences remain even after excluding participants with any neurosensory impairments.

## Results

### Sample description

**Figure 1.**
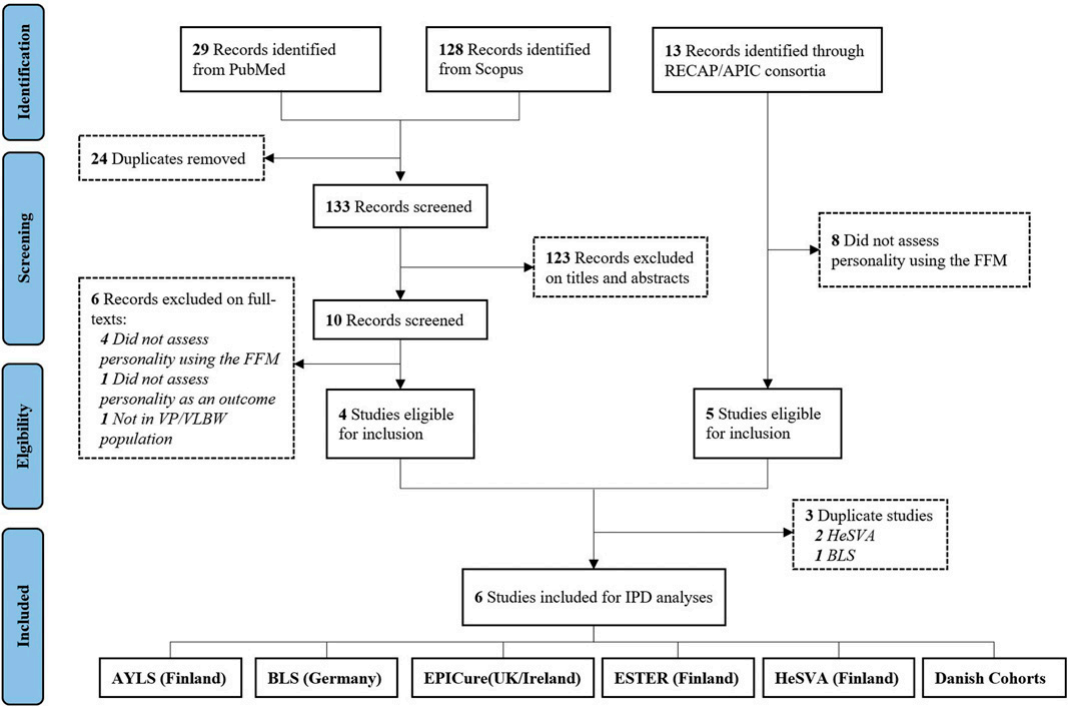
Flowchart of eligible cohorts included in the IPD meta-analysis.

There were 13 studies within the RECAP/APIC consortia, of which five met the inclusion criteria (eight did not assess personality in adulthood in the VP/VLBW population using the Five-Factor Model). A search of the PubMed and Scopus databases identified 133 records (after de-duplication), of which the full texts of 10 records were assessed for eligibility, and four met the inclusion criteria. Six unique studies in total were eligible for inclusion (three were duplicates which appeared in both the RECAP/APIC consortia as well as the literature search) (Figure 1). A description of these studies can be found in Table 1. Of these six studies, three were identified from the RECAP/APIC consortia only: the Arvo Ylppö Longitudinal study (AYLS) (Heinonen et al., 2008; Lano, 2002; Riegel & Betke, 1995), the ESTER preterm birth study (Matinolli et al., 2017; Sipola-Leppänen et al., 2015), and the EPICure study (Marlow et al., 2005; Wood et al., 2000); two were identified from both the RECAP/APIC consortia and the literature search: the Bavarian Longitudinal Study (BLS) (Eryigit-Madzwamuse, Strauss et al., 2015; Wolke & Meyer, 1999), and the Helsinki Study of Very Low Birth Weight Adults (HeSVA) (Hovi et al., 2007; Kaseva et al., 2012; Pesonen et al., 2008). A further study was identified from the literature search only: the Danish 1974–1976 and 1980–1982 cohorts (Hertz et al., 2013). The authors of this Danish study were contacted to obtain individual-level data, however these were not available as the original records were destroyed in accord with data protection; individual data from the Danish registry are never shared outside Denmark, so only published aggregate data were included.

**Table 1.** Description of cohorts included in the IPD meta-analysis.

Source	Cohort	Country	Year of birth	Age of assessment	Type of population	Personality questionnaire used	Initial VP/VLBW surviving to discharge	VP/VLBW eligible in adulthood^a^	VP/VLBW assessed in adulthood	No. of VP/VLBW assessed on personality	No. of term-born participants (>37 weeks/>3,500 g)^b^
RECAP/APIC	AYLS	Finland	1985–1986	25.5	Preterm birth <37 weeks	NEO-FFI (short version of NEO-PI-R) and additional 14 items from NEO-FFI-R	108	68	35	27	305
Eryigit-Madzwamuse; Strauss et al., 2015; RECAP/APIC	BLS	Germany	1985–1986	26	VP (<32 weeks)/VLBW (<1500 g)	BFI-10	510	411	260	200	197
RECAP/APIC	EPICure	UK/Ireland	1995	19	EP (<26 weeks)	BFI-10	315	306	129	115	62
RECAP/APIC	ESTER	Finland	1986	23	Preterm birth <37 weeks	NESTA (adaptation of NEO-PI)	NA	77	77	69	328
Pesonen et al. (2008); Kaseva et al. (2012); RECAP/APIC	HeSVA	Finland	1978–1985	25	VLBW (<1500 g))	NESTA (adaptation of NEO-PI)	334	254	165	157	168
Hertz et al. (2013)	Danish cohort (2 cohorts included)	Denmark	1974–1976; 1980–1982	32–34; 26–28	VP (<32 weeks)	NEO-FFI	NA	1974–1976 (150); 1980–1982 (150)	N/A	1974–1976 (124); 1980–1982 (103)	1974–1976 (104); 1980–1982 (101)

^a^Eligibility defined as participants who were available for follow-up in adulthood.

^b^Term-born classified using 37 weeks gestation as HeSVA and EPICure did not recruit term-born controls at birth; therefore, birth weight data were not available.

*Notes*: BFI-10: The Big Five Inventory-10; IPD = individual participant data; NEO-FFI: NEO Five-Factor Inventory; NA: not applicable; NEO-PI: NEO Personality Inventory; VP/VLBW: very preterm or very low birth weight.

In total, 1,628 participants had data on personality traits in adulthood across the five cohorts with available individual-level data, of which 568 (34.9%) were born VP/VLBW. Both the BLS and EPICure cohorts assessed personality using the BFI-10, using the German and English version, respectively. ESTER and HeSVA used the NESTA (Finnish version of the NEO-PI), where some items were directly translated into Finnish and others replaced with culturally appropriate items (Hämäläinen, 1994). AYLS and the Danish cohort assessed personality using the NEO-FFI, but AYLS contained an additional 14 items taken from the NEO-FFI-R (Aluja et al., 2005). Cronbach’s alpha showed acceptable to excellent internal consistency across the five personality traits in AYLS, ESTER and HeSVA cohorts (α = .63–.94) (see Supplementary Materials, Table S3). As the BLS and EPICure cohorts only assessed each personality trait using two items (apart from agreeableness in EPICure which comprised of 3 items), Pearson correlation coefficient was used to calculate the strength of association between them (Supplementary Materials, Table S4). A weak correlation (*r* < .25) was found for agreeableness in BLS and openness in EPICure; thus, only one of the items (the item which did not require reverse coding) was used rather than calculating the average.

Parental education was assessed in childhood in AYLS, BLS, and EPICure, and in adulthood in ESTER and HeSVA, and all studies used maternal education apart from ESTER which used combined maternal and paternal education. The distribution of parental education level differed between the two groups, with fewer parents of VP/VLBW participants having high educational attainment (Table 2). The distribution of variables in each cohort and the correlation between variables can be found in Supplementary Materials (Tables S5 and S6). Birth weight and gestational age were moderately correlated with each other (lower birth weight correlating with lower gestational age), and delivery mode (caesarean section) and bronchopulmonary dysplasia also showed a small to moderate correlation with birth weight and gestational age (Table S6).

**Table 2.** Chi-squared test of sex, parental education, and age differences between VP/VLBW and term-born participants in all cohorts (*N* = 1,628).

	VP/VLBW (*n* = 568)	Controls (*n* = 1060)	*p*-value
Sex			.223
[Male]	267 (47.0%)	464 (43.7%)	
[Female]	301 (53.0%)	596 (56.3%)	
Parental education			**<.001**
[High, ISCED level 6–8]	94 (16.8%)	354 (33.6%)	
[Medium, ISCED level 3–5]	342 (61.3%)	507 (48.1%)	
[Low, ISCED level 0–2]	122 (21.9%)	192 (18.2%)	
Age of assessment [*M (SD*)]	23.87 (2.90)	24.47 (1.98)	**<.001**

*Notes*. Controls = term-born adults; ISCED: International Standard Classification of Education; VP/VLBW: very preterm or with very low birth weight.

### Critical appraisal of included studies

The Newcastle-Ottawa Scale was used to critically assess the quality of cohort studies. Almost all cohort studies included had a low risk of bias on the selection domain (exposed cohort being representative of the average sample in the community, non-exposed cohort being drawn from the same community, using secure records to ascertain exposure, outcome of interest not being present at the start of the study), apart from the EPICure cohort, as term-born individuals were drawn from a different source to VP/VLBW individuals. All studies were controlled for sex and parental education, and therefore were considered to be high on comparability. Some concerns were raised on the outcome domain, particularly as all studies assessed the outcome using self-report, and some studies (AYLS, BLS, and EPICure) also had a lower rate of follow-up (<50%). Overall, ESTER and HeSVA received the highest scores and EPICure received the lowest.

### Primary analysis (one-stage IPD)

#### Personality differences between VP/VLBW and term-born participants

In comparison with term-born participants, VP/VLBW adults had lower standardized mean differences for extraversion and openness, and higher standardized mean differences for neuroticism and agreeableness (Table 3); no differences between groups were found for conscientiousness. All estimates remained of a similar quantum and significance after adjusting for sex and parental education (Table 3), suggesting that differences in personality between VP/VLBW and term-born participants are not explained by these common sociodemographic factors. No significant interactions between VP/VLBW birth and sex and between VP/VLBW and parental education were found (Supplementary Materials, Table S7). Low heterogeneity was found between studies on age of assessment, year of study recruitment, and levels of attrition, indicating low variance between the cohorts (τ_00_) and larger within-subject variance (σ^2^).

**Table 3.** Standardized mean differences, unadjusted and adjusted for sex, and parental education level, between VP/VLBW and term-born participants for each of the five personality traits (*N* = 1,628).

	Neuroticism	Extraversion	Openness	Agreeableness	Conscientiousness
	β (95% CI)	β (95% CI)	β (95% CI)	β (95% CI)	β (95% CI)
Fixed effect					
VP/VLBW (unadjusted)	**0.23 (0.11, 0.35)**	**−0.42 (-0.53, -0.31)**	**−0.25 (-0.36, -0.14)** ^a^	**0.13 (0.03, 0.23)**	0.08 (−0.05, 0.18)
Random effect					
σ^2^	1.10	1.06	1.11	1.02	1.06
τ_00_ age	0.00	0.00	0.00	0.00	0.01
τ_00_ year	0.02	0.00	0.00	0.00	0.00
τ_00_ dropout	0.00	0.00	0.00	0.00	0.00
Fixed effect					
VP/VLBW (adjusted)	**0.25 (0.14, 0.36)**	**−0.39 (-0.50, -0.28)**	**−0.19 (-0.30, -0.08)** ^b^	**0.17 (0.07, 0.28)**	0.08 (−0.04, 0.18)
Random effect					
σ^2^	1.01	1.05	1.05	1.00	1.05
τ_00_ age	0.00	0.00	0.00	0.00	0.00
τ_00_ year	0.01	0.00	0.00	0.00	0.00
τ_00_ dropout	0.01	0.00	0.00	0.00	0.00

^a^*N* = 1,627.

^b^*N* = 1,610.

*Notes*. VP/VLBW = very preterm or very low birth weight; β = standardized estimates; CI = confidence interval; σ^2^ = residual variance/within-subject variance; τ_00_ = random intercept variance/between-subject variance; Age = age of assessment; Year = year of recruitment; Dropout = levels of attrition.

#### Subgroup analyses: Neonatal characteristics

Within the VP/VLBW population, after adjusting for sex and maternal education, individuals with neonatal bronchopulmonary dysplasia had statistically significantly higher scores for neuroticism and lower scores for extraversion compared to those without (Table 4). No effects of gestational age and delivery mode and no significant differences in openness, agreeableness, or conscientiousness were found (Table 4).

**Table 4.** Standardized mean differences of individual predictors for each of the five personality traits within the VP/VLBW Population (*N* = 568).

Fixed effect^a^	Neuroticism	Extraversion	Openness	Agreeableness	Conscientiousness
	*β (95% CI)*	*β (95% CI)*	*β (95% CI)*	*β (95% CI)*	*β (95% CI)*
Gestational age	0.02 (−0.04, 0.07)	0.01 (−0.04, 0.06)	0.01 (−0.04, 0.06)	0.02 (−0.02, 0.06)	0.03 (−0.02, 0.08)
Birth weight z score	0.05 (−0.04, 0.15)	0.02 (−0.08, 0.11)	0.03 (−0.07, 0.13)	0.06 (−0.03, 0.15)	0.09 (−0.01, 0.19)
Delivery mode [ref: vaginal]	0.08 (−0.13, 0.29)	0.05 (−0.16, 0.26)	0.03 (−0.19, 0.25)	−0.05 (−0.25, 0.15)	−0.05 (−0.26, 0.16)
Bronchopulmonary dysplasia [ref: none]	**0.24 (0.04, 0.45)**	**−0.25 (-0.46, -0.03)**	−0.07 (−0.29, 0.14)	−0.01 (−0.20, 0.17)	−0.07 (−0.28, 0.14)
Random effect					
σ^2^	1.14	1.16	1.27	1.05	1.15
τ_00_ age	0.03	0.01	0.01	0.00	0.02
τ_00_ year	0.02	0.01	0.00	0.00	0.01
τ_00_ dropout	0.04	0.01	0.01	0.00	0.02

^**a**^Adjusted for sex and parental education levels.

*Notes*. VP/VLBW = very preterm or very low birth weight; β = standardized estimates; CI = confidence interval; σ^2^ = residual variance/within-subject variance; τ_00_ = random intercept variance/between-subject variance; Age = age of assessment; year = year of recruitment; Dropout = level of attrition.

#### Subgroup analyses: VP/VLBW participants without neurosensory impairments

All analyses were repeated after exclusion of VP/VLBW participants who had any neurosensory impairments in childhood. Findings remained consistent with similar effect sizes to those described above, suggesting that personality differences between VP/VLBW and term-born controls, as well as the association between bronchopulmonary dysplasia and personality differences within the VP/VLBW population, are not explained by severe neurological insult associated with moderate to severe functional limitations in VP/VLBW participants in childhood (Supplementary Materials, Table S8; Table 5).

**Table 5.** Standardized mean differences of individual predictors for each of the five personality traits within the VP/VLBW population (after removing participants with neurosensory impairments) (*N* = 479).

Fixed effect^a^	Neuroticism	Extraversion	Openness	Agreeableness	Conscientiousness
	*β (95% CI)*	*β (95% CI)*	*β (95% CI)*	*β (95% CI)*	*β (95% CI)*
Gestational age	0.03 (−0.03, 0.09)	0.02 (−0.03, 0.08)	0.03 (−0.03, 0.08)	0.03 (−0.02, 0.07)	0.02 (−0.04, 0.07)
Birth weight z score	0.05 (−0.06, 0.15)	0.05 (−0.05, 0.16)	0.04 (−0.07, 0.15)	0.07 (−0.03, 0.16)	0.08 (−0.02, 0.19)
Delivery mode [ref: vaginal]	0.07 (−0.16, 0.29)	0.04 (−0.20, 0.27)	0.01 (−0.22, 0.25)	−0.12 (−0.33, 0.09)	−0.05 (−0.27, 0.18)
Bronchopulmonary dysplasia [ref: none]	**0.26 (0.04, 0.49)**	**−0.30 (-0.53, -0.07)**	−0.00 (−0.23, 0.23)	−0.02 (−0.22, 0.19)	−0.10 (−0.32, 0.13)
Random effect					
σ^2^	1.07	1.17	1.20	1.01	1.09
τ_00_ age	0.05	0.00	0.01	0.00	0.02
τ_00_ year	0.04	0.02	0.01	0.00	0.02
τ_00_ dropout	0.05	0.02	0.01	0.00	0.01

^**a**^Adjusted for sex and parental education levels.

*Notes*. VP/VLBW = very preterm or very low birth weight; β = standardized estimates; CI = confidence interval; σ^2^ = residual variance/within-subject variance; τ_00_ = random intercept variance/between-subject variance; Age = age of assessment; Year = year of recruitment; Dropout = level of attrition.

### Secondary analysis (two-stage IPD)

**Figure 2.**
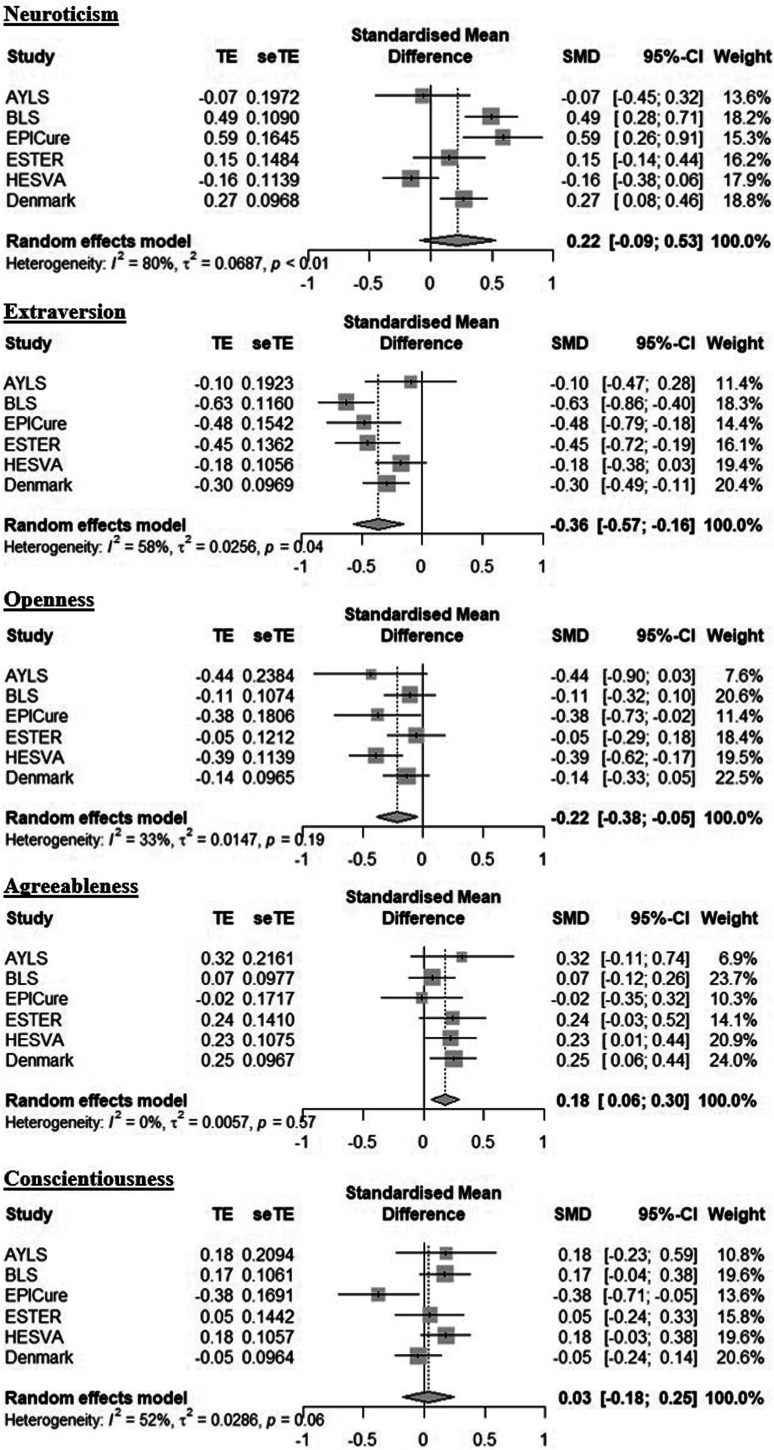
Secondary analysis: Forest plots showing personality differences between VP/VLBW and term-born participants, including aggregate data from the Danish cohort.

A two-stage IPD meta-analysis was conducted by combining effect sizes from each cohort, including aggregate data from the Danish study. Two sets of published estimates from the Danish study were available as two cohorts were included in the study; however, the published paper also presented estimates for both cohorts combined (Hertz et al., 2013). These combined estimates (mean *t*-scores) from the two cohorts were used in this secondary analysis and transformed into standardized mean differences (Lipsey & Wilson, 2001; Wilson, 2017, 2023). Results were similar to the one-stage approach: VP/VLBW adults scored lower on extraversion and openness and higher on agreeableness than term-born participants (Figure 2). The effect size for neuroticism was similar compared to the one-stage approach, but this did not reach statistical significance tested at *p* < .05 in the two-stage approach. There was considerable heterogeneity between the studies on neuroticism (I^2^ = 80%). This was different to the measures of heterogeneity reported from the one-stage analysis, which may be explained by the different measures of heterogeneity used, and that although there is little variability among the effect sizes between studies (indicated by low τ value), there may be considerable variation between studies that is not explained by chance alone (indicated by large I^2^) (Huang, 2023; Steenbergen, 2020).

### Robustness analyses

#### Bronchopulmonary dysplasia defined as >28 days supplemental oxygen after birth

We repeated analyses within the VP/VLBW population after removing one cohort (EPICure) which used a different definition of bronchopulmonary dysplasia. Neonatal bronchopulmonary dysplasia (defined only as supplemental oxygen use for more than 28 days after birth) was still associated with lower scores on extraversion, with similar effect sizes compared to when all cohorts were included, although its effect on neuroticism was weakened and no longer statistically significant (Supplementary Materials, Table S9). Findings remained the same after removing VP/VLBW participants with childhood neurosensory impairments (Supplementary Materials, Table S10).

#### Two-stage IPD using only cohorts included in one-stage

A forest plot showing the findings from a two-stage IPD using only cohorts included in the one-stage IPD analysis can also be found in Supplementary Materials (Figure S1), with similar effects as those reported in primary and secondary analyses.

## Discussion

The aim of this study was to examine personality differences in the VP/VLBW population compared to term-born adults, and to investigate whether neonatal characteristics and childhood neurosensory impairments may help explain variations in personality traits within the VP/VLBW population. Findings from five cohorts showed that VP/VLBW adults scored lower on extraversion and openness and higher on neuroticism and agreeableness compared to term-born participants. The magnitude of these differences remained similar after accounting for sex and parental education, and no interactions were found with them, suggesting that sex and parental education—two important predictors of personality differences in the literature (Schmitt et al., 2008; Sutin et al., 2017)—did not differentially influence the association of VP/VLBW birth on personality. Further analyses within the VP/VLBW population discovered that those with a history of bronchopulmonary dysplasia scored lower on extraversion and higher on neuroticism even after removing participants with a history of neurosensory impairments in childhood. Altogether, these findings tentatively suggest that personality differences between VP/VLBW and term-born adults, as well as individual differences within the VP/VLBW population, may be partly due to neonatal morbidities and brain injury arising from VP/VLBW birth, rather than sociodemographic (i.e., maternal education) or functional limitations investigated in this current study.

The findings that VP/VLBW adults scored lower on extraversion and higher on neuroticism are consistent with the existing literature (Allin et al., 2006; Eryigit-Madzwamuse, Strauss et al., 2015; Hertz et al., 2013; Lyall et al., 2016; Schmidt et al., 2008; Waxman et al., 2013). This adds further support to the previously defined preterm personality phenotype, which is characterized by shyness, social withdrawal, less risk-taking behaviors, and increased internalizing symptoms in childhood and adulthood (Johnson et al., 2011, 2018; Pyhälä et al., 2017; Wolke et al., 2019). The finding that VP/VLBW adults scored higher on agreeableness is also consistent with a few studies (Eryigit-Madzwamuse, Strauss et al., 2015; Hertz et al., 2013), albeit somewhat surprising given that agreeableness has been associated with higher IQ scores (Bartels et al., 2012) whereas VP/VLBW birth is associated with lower IQ (Eryigit-Madzwamuse, Baumann et al., 2015). It has been postulated that agreeableness may originate from early relationship and attachment with the caregiver (Graziano & Eisenberg, 1997). Whereas this may be disturbed in infants looked after in a neonatal intensive care unit, mothers of VP/VLBW children may not be any less sensitive or responsive compared to mothers of term-born children (Bilgin & Wolke, 2015). Furthermore, agreeableness has been correlated negatively with externalizing problems such as hostility (Miller et al., 2008). VP/VLBW adults consistently report more internalizing symptoms and lower externalizing symptoms than term-born (Johnson & Marlow, 2011), which may explain their higher agreeableness scores compared to term-born adults. VP/VLBW adults also scored lower on openness, consistent with one previous study (Pesonen et al., 2008), although no differences were found for conscientiousness.

Findings of personality differences between VP/VLBW and term-born adults may also be interpreted within a biological framework of personality. Eysenck (1963) and later Gray (1987) suggested that neuroticism and extraversion may be associated with the activation of different neural or hormone modulatory systems (DeYoung & Blain, 2020). Neuroticism has been consistently associated with the activity of amygdala and hippocampus (DeYoung & Blain, 2020; Garcia-Banda et al., 2014; Holmes et al., 2012; Kaczkurkin et al., 2016; Schuyler et al., 2014; Servaas et al., 2013), as well as alterations in hypothalamic–pituitary–adrenal (HPA) axis function, including the levels of the stress hormone cortisol, but often with inconsistent and contradictory findings due to differences in experimental design or heterogeneity in the measures used (Ormel et al., 2013). Extraversion, on the other hand, is theorized to be associated with cortical arousal levels (Eysenck, 1963; Gray, 1987; Pickering & Gray, 1999), as well as with brain regions implicated in reward processing, including the medial orbitofrontal cortex and striatum (Barrós-Loscertales et al., 2006; DeYoung et al., 2010; Wu et al., 2014). Less evidence exists for neurobiological underpinning for the other three personality traits. Openness has been associated with cognitive abilities and dompaminergic function as well as differential patterns of white matter connectivity (DeYoung, 2013, 2020; Jung et al., 2010) whereas agreeableness has been found to relate to regions responsible for social information processing and empathy, such as the medial prefrontal cortex and mirror neuron system (DeYoung & Blain, 2020; Rizzolatti, 2005; Seitz et al., 2006). Finally, the primary neurobiological basis of conscientiousness has been linked with the prefrontal cortex which is involved in cognitive processing of goals and the ability to plan and follow complex rules (DeYoung & Blain, 2020; Kapogiannis et al., 2013; Riccelli et al., 2017). However, these biological underpinnings of personality have received mixed support in the literature, as sample sizes in neuroimaging studies have been small, and recent evidence suggests that the association between brain structure and personality may be partly attributed to genetic differences instead, with approximately 40% of variations in personality found to be heritable (Vukasović & Bratko, 2015).

Nevertheless, the finding that specific neonatal factors within the VP/VLBW population partly explained variations in personality may contribute further evidence to the biological framework of personality, which requires further investigation. For example, neonatal bronchopulmonary dysplasia was associated with lower extraversion and higher neuroticism scores within the VP/VLBW population. Bronchopulmonary dysplasia is a chronic lung condition which mostly affects infants born very preterm who require mechanical ventilation with oxygen supplementation (Kinsella et al., 2006). VP/VLBW infants with bronchopulmonary dysplasia have smaller brain volumes and altered white matter characteristics compared to those without BPD, suggesting a particular association with disturbed brain development (Lee et al., 2019; Neubauer et al., 2015). Thus, one potential explanation for the association of bronchopulmonary dysplasia with neuroticism and extraversion might be through its effect as a marker of more severe neonatal conditions, which are more likely to cause alterations in brain development (Kelly et al., 2023). There is more support in the literature for a biological origin of these two personality traits compared to the other three traits of the Five-Factor Model (DeYoung & Blain, 2020), which is consistent with findings of this study, where bronchopulmonary dysplasia was only associated with variations in neuroticism and extraversion traits. Furthermore, the effects of bronchopulmonary dysplasia persisted when children with the most severe functional limitations were removed. This further suggests that the effect of bronchopulmonary dysplasia on personality may be partly attributed to disturbed brain development rather than other functional impairments resulting from this neonatal complication (Neubauer et al., 2015; Schmidt, 2003; Siffel et al., 2021), but further research is needed to rule out the presence of other factors such as genetics.

Altogether, these findings suggest that at least part of the differences in personality observed in the VP/VLBW population may be interpreted and explained using a biological framework of personality (DeYoung & Blain, 2020; Eysenck, 1963; Zuckerman, 2012). Although we did not test specific biological mechanisms underlying personality traits, given that there is consistent evidence for frequent brain injuries and neurological developmental disturbances in those born VP/VLBW (Bäuml et al., 2015; Meng et al., 2016; Volpe, 2009), it may be inferred that part of the personality differences observed in this population may be attributed to these biological risks, which persisted after accounting for sex and parental education, and excluding those with severe disabling conditions (i.e., neurosensory impairments) in childhood. However, only sex and parental education were considered as confounders in the study due to the availability of data across studies, but there may be other confounders that may explain some of the personality differences in the VP/VLBW population. For example, prenatal maternal depression has been associated with increased risk of preterm birth and low birth weight, possibly due to biological changes from the release of stress hormones which can restrict nutrients to the fetus, and lead to growth restriction or early delivery (Grote et al., 2010). Maternal depression has also been associated with suboptimal parent-child relationship which may affect child temperament (Hazell Raine et al., 2020). The combination of biological changes and parenting behavior as a result of maternal depression may therefore also influence the development of personality. However, there is no evidence to suggest that parenting behaviors differ between VP/VLBW and term-born controls (Bilgin & Wolke, 2015), although further research is needed to examine possible biological pathway as well. Furthermore, a new large sample prospective research that started before pregnancies resulting in preterm birth indicated no differences in life satisfaction of parents before and during pregnancy. Rather, preterm birth reduced life satisfactions in the first two years after birth for mothers (Eves et al., 2023). There may also be a genetic component, given the moderate heritability of personality (Bleidorn et al., 2016; Sanchez-Roige et al., 2018) and the identification of genetic predictors for preterm birth (Mead et al., 2023). These were not investigated in this study due to the lack of genetic data.

Although this study cannot exclude the effect of other confounders, the synthesis of evidence across six European VP/VLBW cohorts has contributed further evidence on personality differences in domains that have not been examined much previously or have received mixed findings (i.e., openness and conscientiousness), as well as domains which may have biological correlates (i.e., extraversion and neuroticism were the only personality domains significantly associated with bronchopulmonary dysplasia). Personality is implicated in many areas of adult functioning, including health, education, and social relationships (Roberts et al., 2007). Hence, understanding personality differences in those born VP/VLBW may help explain the association between VP/VLBW birth and altered functioning in these areas, which have been frequently reported in the literature. For example, lower extraversion scores have been associated with smaller social networks and reduced social support (Zhu et al., 2013), which may explain why overall VP/VLBW adults also report fewer romantic relationships (Mendonça et al., 2019) or friends (Ni et al., 2021). Similarly, some have suggested a causal relationship between neuroticism and depression (Jeronimus et al., 2016; Speed et al., 2019), and VP/VLBW birth is also associated with increased risk of psychiatric disorders such as depression and anxiety (Anderson et al., 2021; Fitzallen et al., 2021; Nosarti et al., 2012; Robinson et al., 2020). Personality may thus be an important mediator in other life outcomes for the VP/VLBW population, and it would be important for future studies to combine neurobiological, psychosocial, and genetic data within the VP/VLBW population, which may also have implications for understanding personality development in the general population.

### Strengths and limitations of the study

There are several strengths to this IPD meta-analysis. First, the inclusion of individual-level data allowed for a large sample size to maximize power for testing of differences in personality scores between VP/VLBW adults and term-born participants. Access to individual-level data also enabled more detailed investigation of other predictors beyond initial group differences, which is often limited in traditional meta-analysis with aggregate data (Burke et al., 2017). Second, the eligibility criteria ensured that only studies which examined personality using the Five-Factor Model were included. This helped to ensure that similar personality traits were measured across different studies. Third, a secondary analysis using aggregate data from another cohort (Danish cohort) identified from the literature revealed similar findings despite the differences in statistical approach (one-stage vs two-stage).

Our study also has some limitations. First, despite only including cohorts which have assessed personality using the Five-Factor Model, several personality questionnaires (i.e., the BFI-10 and variations of NEO-PI) in different languages were used. Although all cohorts assessed personality across five traits, the used instruments differ in their definition and measurement of the personality traits. For instance, previous research has shown that the correlations between the BFI-10 and NEO-PI respective factors are relatively low for openness and agreeableness due to the reduction of items and loss of information (Rammstedt & John, 2007; Schmitt et al., 2007). However, little heterogeneity was found between studies on these two personality traits, indicating that findings were quite homogenous regardless of the type of instrument used across studies. Second, data came exclusively from European countries (i.e., UK/Ireland, Germany, Finland, and Denmark). It has been previously shown that there may be some culture-specific personality differences (Allik et al., 2017), in particular for sex differences which are larger in North America and Europe than in Eastern/South Eastern Asia (Schmitt et al., 2008); thus, results may not be generalizable to other settings. Third, only a small selection of predictors and confounders was considered in this study due to the availability of data across cohorts. Personality traits have been previously found to have a moderate genetic basis (Sanchez-Roige et al., 2018; Vukasović & Bratko, 2015), and prenatal stress such as maternal depression may also contribute to preterm birth via biological processes which may also be associated with differences in personality. These data were not available and could not be considered in the study. Different definitions for bronchopulmonary dysplasia were used in the study. This was a limitation of harmonizing data across cohorts which have assessed bronchopulmonary dysplasia in different ways. However, we repeated the analyses by including only cohorts which used the mild definition of bronchopulmonary dysplasia, and similar findings emerged. Lastly, some deviations from the pre-registration completed before data were available should be noted: secondary outcomes (risk-taking and delinquent behaviors) were not investigated given that data agreements were not completed before the end of the study. Although this was not intentional, these outcomes may also receive less attention in the literature, which may contribute further to publication bias. Sex was considered as a covariate rather than a predictor, which doesn’t change the results of the analyses, but instead shifts the interpretation of the results from explaining the effect of sex on personality, to whether personality differences between VP/VLBW and term-born adults remained after controlling for sex. We examined the role of neurosensory impairments by comparing findings before and after removing those with neurosensory impairments. We conducted this analysis rather than utilizing neurosensory impairment as a mediator/moderator due to the fact that these impairments are widely defined as cerebral palsy or visual or hearing impairment or cognitive disability. These impairments refer to different, often neurological origins, were not assessed the same across cohorts and thus we opted to exclude this heterogenous group instead in sensitivity analysis. Results remained very similar; however, as we did not examine the moderating role of neurosensory impairments, no conclusions can be drawn on this research question.

## Conclusion

Using individual-level longitudinal data from five European cohorts, we found consistent evidence of VP/VLBW adults scoring higher on neuroticism and agreeableness and lower on extraversion and openness to new experiences than their term-born peers. Further analyses within the VP/VLBW population found that the presence of bronchopulmonary dysplasia—an indicator of more severe neonatal complications and proxy measure for altered brain development—was further associated with individual differences in extraversion and neuroticism, even after excluding those with severe functional limitations in childhood, suggesting stronger biological underpinnings to these personality traits, although other confounders such as prenatal maternal psychopathology and genetics may also play a role. Future research should investigate whether alterations in brain development—as assessed with neuroimaging studies—mediate the association between VP/VLBW birth and personality, in particular, for neuroticism and extraversion.

## Supplemental Material

Supplemental Material - The effect of very preterm birth on the five-factor model of personality traits: A meta-analysis of individual participant data Supplemental Material for The effect of very preterm birth on the five-factor model of personality traits: A meta-analysis of individual participant data by Yiwen Liu, Anu Realo, Marina Mendonça, Nicole Baumann, Peter Bartmann, Katri Räikkönen, Kati Heinonen, Rachel Robinson, Neil Marlow, Samantha Johnson, Yanyan Ni, Eero Kajantie, Petteri Hovi, Marjaana Tikanmäki and Dieter Wolke in European Journal of Personality.

Supplemental Material - The effect of very preterm birth on the five-factor model of personality traits: A meta-analysis of individual participant data Supplemental Material for The effect of very preterm birth on the five-factor model of personality traits: A meta-analysis of individual participant data by Yiwen Liu, Anu Realo, Marina Mendonça, Nicole Baumann, Peter Bartmann, Katri Räikkönen, Kati Heinonen, Rachel Robinson, Neil Marlow, Samantha Johnson, Yanyan Ni, Eero Kajantie, Petteri Hovi, Marjaana Tikanmäki and Dieter Wolke in European Journal of Personality.
