# Supplementary material for: The effect of very preterm birth on the Five-Factor Model of personality traits: A meta-analysis of individual participant data
Source: Eur J Pers. 2024 Oct 24;39(4):498–517. doi: 10.1177/08902070241280101 (PMC13029488; doi:10.1177/08902070241280101)
Supplement: Supplemental Material - The effect of very preterm birth on the five-factor model of personality traits: A meta-analysis of individual participant data [file sj-pdf-1-erp-10.1177_08902070241280101.pdf]

Supplementary materials

Table S1. Newcastle-Ottawa Scale ratings for each cohort

Cohort	Selection				Comparability	Outcome			Total
	Representative-ness of exposed cohort (★ = truly representative of the average in the community)	Selection of non-exposed cohort (★ = drawn from same community as exposed cohort)	Ascertainment of exposure (★ = secure record)	Demonstration that outcome of interest was not present at start of study (★ = Yes)	Comparability of cohorts on the basis of the design or analysis (★★ = controls for sex and parental education)	Assessment of outcome	Was follow-up long enough for outcomes to occur (★ = Yes; ≥18 years)	Adequacy of follow up of cohorts[a] (★ = follow-up rate >50%)	
AYLS	★	★	★	★	★★	self-report	★	Follow-up <50%	★★★★★★★
BLS	★	★	★	★	★★	self-report	★	Follow-up <50%	★★★★★★★
EPICure	★	Different source	★	★	★★	self-report	★	Follow-up <50%	★★★★★★
ESTER	★	★	★	★	★★	self-report	★	★	★★★★★★★★
HeSVA	★	★	★	★	★★	self-report	★	★	★★★★★★★★

[a]Based on % of VP/VLBW participants eligible and assessed in adulthood

Table S2. Number of missing cases imputed for VP/VLBW participants in each cohort.

**Predictors**	**AYLS**	**BLS**	**EPICure**	**ESTER**	**HeSVA**
Parental education	-	3	6	-	1
Delivery mode	3	37	1	-	1
BPD	26	-	-	29	6

Table S3. Internal consistency reliability (Cronbach's alpha) of the personality domains in AYLS, ESTER and HeSVA

Cohort	Neuroticism	Neuroticism	Openness	Agreeableness	Conscientiousness
AYLS	0.90	0.90	0.75	0.79	0.87
ESTER	0.94	0.94	0.88	0.71	0.85
HeSVA	0.94	0.94	0.90	0.63	0.87

Note. AYLS = Arvo Ylppö Longitudinal study, N=332, assessed personality using the NEO-FFI and NEO-FFI-R; ESTER = ESTER preterm birth study, N=397, assessed personality using the NEO-PI; HeSVA = Helsinki Study of Very Low Birth Weight Adults, N=325, assessed personality using the NEO-PI.

Table S4. Pearson's correlation between items on each personality domain in the BLS and EPICure cohorts

Cohort	Neuroticism	Extraversion	Openness	Agreeableness	Conscientiousness
BLS	0.37 [b]	0.54[b]	0.38 [b]	-0.02	0.40 [b]
EPICure	0.43[b]	0.37[b]	-0.10	0.50[a]	0.31[b]

[a] Cronbach's alpha was calculated as there were three items on this domain
[b] $p<0.01$
Note. BLS = Bavarian Longitudinal Study, N=397, assessed personality using the BFI-10; EPICure = EPICure study, N=177, assessed personality using the BFI-10.

Table S5. Distribution of variables in the VP/VLBW population and term-born participants

	**AYLS**		**BLS**		**EPICure**		**ESTER**		**HESVA**	
	VP/VLBW (N=27)	Term-born (N=305)	VP/VLBW (N=200)	Term-born (N=197)	VP/VLBW (N=115)	Term-born (N=62)	VP/VLBW (N=69)	Term-born (N=328)	VP/VLBW (N=157)	Term-born (N=168)
	N (%)									
Sex [Female]	12 (44.4%)	176 (57.7%)	95 (47.5%)	103 (52.3%)	64 (55.7%)	39 (62.9%)	40 (58.0%)	176 (53.8%)	90 (57.3%)	102 (60.7%)
Parental education										
[High]	12 (44.4%)	151 (49.8%)	27 (13.7%)	32 (16.3%)	4 (3.7%)	12 (19.4%)	22 (31.9%)	108 (33.2%)	29 (18.6%)	51 (30.5%)
[Medium]	8 (29.6%)	97 (32.0%)	109 (55.3%)	76 (38.8%)	84 (77.1%)	47 (75.8%)	43 (62.3%)	197 (60.6%)	98 (62.8%)	90 (53.9%)
[Low]	7 (25.9%)	55 (18.2%)	61 (31.0%)	88 (44.9%)	21 (19.3%)	3 (4.8%)	4 (5.8%)	20 (6.2%)	29 (18.6%)	26 (15.6%)
Delivery mode [Caesarean]	11 (45.8%)	39 (13.2%)	106 (65%)	30 (15.7%)	19 (16.7%)	-	50 (72.5%)	39 (11.9%)	100 (64.1%)	18 (10.7%)
BPD	1 (3.7%)	-	100 (50%)	-	82 (71.3%)	-	14 (35%)	-	29 (19.2%)	-
Multiple birth	3 (11.1%)	4 (1.3%)	54 (27.0%)	7 (3.6%)	39 (34.2%)	-	21 (30.4%)	4 (1.2%)	25 (15.9%)	0
NSI	5 (18.5%)	3 (1.0%)	34 (17.1%)	1 (0.5%)	22 (19.5%)	-	7 (10.1%)	1 (0.3%)	17 (10.9%)	1 (0.6%)
	Mean (SD)									
Age of assessment	25.72 (0.55)	25.43 (0.59)	26.18 (0.59)	26.12 (0.52)	19.28 (0.54)	19.19 (0.54)	23.03 (1.32)	23.55 (1.11)	24.57 (2.03)	24.56 (2.18)
Gestational age	28.96 (2.30)	39.69 (1.17)	30.43 (2.06)	39.67 (1.16)	24.49 (0.73)	-	30.64 (2.04)	40.06 (1.21)	29.18 (2.18)	40.13 (1.15)
Birth weight z-score	0.15 (0.86)	0.15 (0.99)	-0.61 (1.21)	-0.35 (0.85)	0.23 (0.81)	-	0.07 (1.18)	0.08 (0.93)	-0.41 (0.97)	0.10 (0.94)

Note. BPD = Bronchopulmonary dysplasia; NSI = Neurosensory impairment.

Table S6. Correlation matrix showing the association between covariates in the VP/VLBW population across all cohorts (N=568)

	1	2	3	4	5	6
**1. Gestational age**	-					
**2. Birth weight z score**	-0.50*	-				
**3. Sex**	0.01	-0.11*	-			
**4. Parental education**	-0.06	-0.03	0.04	-		
**5. Delivery mode**	0.44*	-0.27*	-0.03	-0.10*	-	
**6. BPD**	-0.31*	0.10*	-0.08	0.05	-0.14*	-

* $p<0.05$

Table S7. Standardised mean differences showing the interaction effect between VP/VLBW and sex, and VP/VLBW and parental education, on differences in FFM personality traits (*N*=1,628)

	**N**	**E**	**O**[a]	**A**	**C**
**Interaction effect**	β (95% CI)	β (95% CI)	β (95% CI)	β (95% CI)	β (95% CI)
VP/VLBW x sex	0.08 (-0.13, 0.29)	-0.07 (-0.29, 0.14)	-0.02 (-0.23, 0.19)	-0.02 (-0.23, 0.18)	-0.11 (-0.32, 0.10)
VP/VLBW x parental education (Medium)	-0.09 (-0.35, 0.18)	0.11 (-0.16, 0.38)	-0.07 (-0.34, 0.21)	-0.14 (-0.41, 0.12)	0.24 (-0.04, 0.51)
VP/VLBW x parental education (Low)	0.09 (-0.24, 0.41)	-0.08 (-0.41, 0.25)	-0.04 (-0.37, 0.29)	-0.20 (-0.52, 0.12)	0.26 (-0.07, 0.59)
**Random effect**					
σ^2	1.01	1.05	1.05	1.00	1.05
τ_{00} Age	0.01	0.00	0.00	0.00	0.00
τ_{00} Year	0.01	0.00	0.00	0.00	0.01
τ_{00} Dropout	0.01	0.00	0.00	0.00	0.01

[a] N=1,627
[b] Effects adjusted for each other
Note. β = standardised estimates; CI = confidence interval; σ^2 = Residual variance/within-subject variance; τ_{00} = Random intercept variance/between-subject variance; Age = Age of assessment; Year = Year of recruitment; Dropout = Levels of attrition.

Table S8. Unadjusted standardised mean differences between VP/VLBW and term-born participants on FFM personality traits, after removing participants with any childhood NSIs (*N*=1533).

	**Unadjusted**				
	**Neuroticism**	**Extraversion**	**Openness**	**Agreeableness**	**Conscientiousness**
**Fixed effect**	β (95% CI)	β (95% CI)	β (95% CI)	β (95% CI)	β (95% CI)
VP/VLBW	**0.23 (0.11, 0.35)**	**-0.43 (-0.54, -0.31)**	**-0.22 (-0.34, -0.11)**	**0.16 (0.05, 0.27)**	0.05 (-0.07, 0.17)
**Random effect**					
σ^2	1.07	1.07	1.07	1.01	1.04
τ_{00} Age	0.00	0.00	0.00	0.00	0.00
τ_{00} Year	0.02	0.00	0.00	0.00	0.01
τ_{00} Dropout	0.00	0.00	0.00	0.00	0.00

Note. β = standardised estimates; CI = confidence interval; σ^2 = Residual variance/within-subject variance; τ_{00} = Random intercept variance/between-subject variance; Age = Age of assessment; Year = Year of recruitment; Dropout = Levels of attrition.

Table S9. Effect of individual predictors on differences in FFM personality traits within the VP/VLBW population (using BPD definition of >28 days only. *N*=453)

	**Neuroticism**	**Extraversion**	**Openness**	**Agreeableness**	**Conscientiousness**
**Fixed effect**[a]	*β (95% CI)*	*β (95% CI)*	*β (95% CI)*	*β (95% CI)*	*β (95% CI)*
Gestational age	0.04 (-0.02, 0.10)	0.01 (-0.05, 0.07)	0.01 (-0.05, 0.07)	0.00 (-0.05, 0.05)	0.00 (-0.06, 0.05)
Birth weight z score	0.08 (-0.03, 0.19)	0.02 (-0.09, 0.13)	0.03 (-0.08, 0.14)	0.01 (-0.08, 0.11)	0.03 (-0.07, 0.13)
Delivery mode [Ref: vaginal]	0.03 (-0.19, 0.26)	0.07 (-0.17, 0.30)	0.02 (-0.21, 0.24)	-0.05 (-0.25, 0.15)	-0.15 (-0.37, 0.07)
BPD [Ref: no BPD]	0.18 (-0.05, 0.42)	**-0.28 (-0.53, -0.04)**	-0.05 (-0.28, 0.18)	0.03 (-0.17, 0.24)	-0.08 (-0.13, 0.30)
**Random effect**					
σ^2	1.14	1.23	1.11	0.94	1.06
τ_{00} Age	0.01	0.01	0.01	0.00	0.00
τ_{00} Year	0.03	0.01	0.01	0.00	0.00
τ_{00} Dropout	0.03	0.01	0.01	0.00	0.00

[a]Adjusted for sex and parental education levels.

Note. All predictors are adjusted for each other. Abbreviations: β = standardised estimates; CI = confidence interval; N = Neuroticism; E = Extraversion; O = Openness; A = Agreeableness; C = Conscientiousness; SGA = Small for gestational age; AGA = appropriate for gestational age; LGA = large for gestational age; BPD = bronchopulmonary dysplasia; NSI = Neurosensory impairment; σ^2 = Residual variance/within-subject variance; τ_{00} = Random intercept variance/between-subject variance; Age = Age of assessment; Year = Year of recruitment; Dropout = Level of attrition.

Table S10. Effect of individual predictors on differences in FFM personality traits within the VP/VLBW population after removing participants with NSIs (using BPD definition of >28 days only. *N*=388)

	**Neuroticism**	**Extraversion**	**Openness**	**Agreeableness**	**Conscientiousness**
**Fixed effect**[a]	*β (95% CI)*	*β (95% CI)*	*β (95% CI)*	*β (95% CI)*	*β (95% CI)*
Gestational age	0.05 (-0.01, 0.12)	0.02 (-0.04, 0.09)	0.013(-0.04, 0.09)	0.00 (-0.05, 0.06)	-0.02 (-0.08, 0.03)
Birth weight z score	0.06 (-0.05, 0.18)	0.07 (-0.06, 0.19)	0.06 (-0.06, 0.17)	0.01 (-0.09, 0.11)	0.02 (-0.09, 0.13)
Delivery mode [Ref: vaginal]	0.06 (-0.18, 0.30)	0.03 (-0.23, 0.29)	-0.02 (-0.26, 0.22)	-0.11 (-0.33, 0.11)	-0.12 (-0.35, 0.12)
BPD [Ref: no BPD]	0.20 (-0.04, 0.45)	**-0.32 (-0.58, -0.05)**	-0.02 (-0.23, 0.27)	0.05 (-0.16, 0.27)	0.02 (-0.21, 0.25)
**Random effect**					
σ^2	1.09	1.23	1.05	0.90	1.00
τ_{00} Age	0.02	0.02	0.01	0.00	0.00
τ_{00} Year	0.02	0.02	0.02	0.00	0.00
τ_{00} Dropout	0.03	0.02	0.01	0.00	0.00

[a]Adjusted for sex and parental education levels.

Note. All predictors are adjusted for each other. Abbreviations: β = standardised estimates; CI = confidence interval; N = Neuroticism; E = Extraversion; O = Openness; A = Agreeableness; C = Conscientiousness; SGA = Small for gestational age; AGA = appropriate for gestational age; LGA = large for gestational age; BPD = bronchopulmonary dysplasia; NSI = Neurosensory impairment; σ^2 = Residual variance/within-subject variance; τ_{00} = Random intercept variance/between-subject variance; Age = Age of assessment; Year = Year of recruitment; Dropout = Level of attrition.

Neuroticism

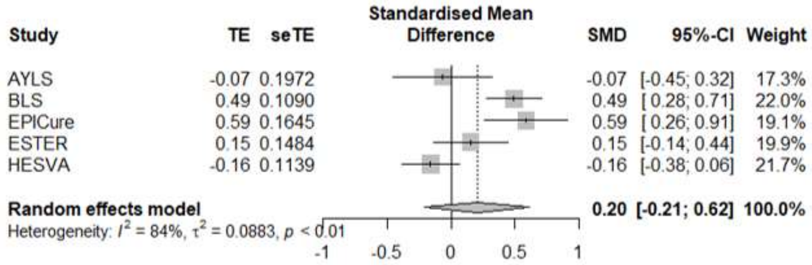

Extraversion

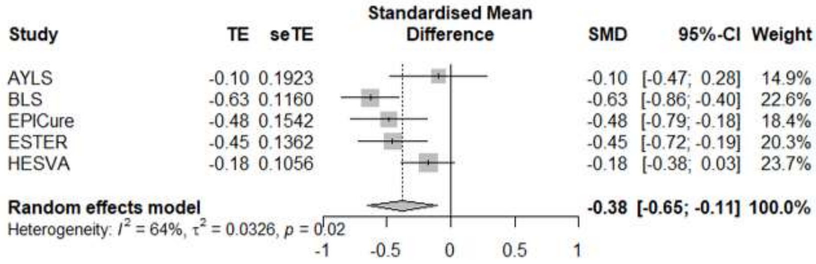

Openness

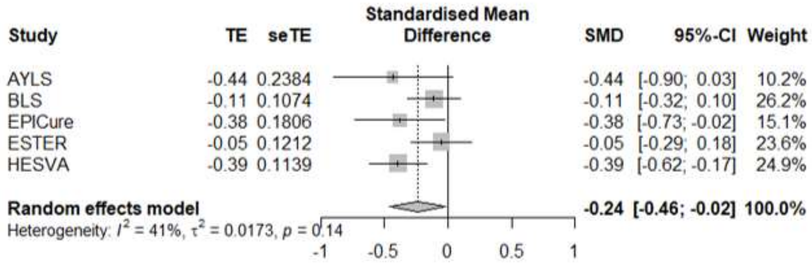

Agreeableness

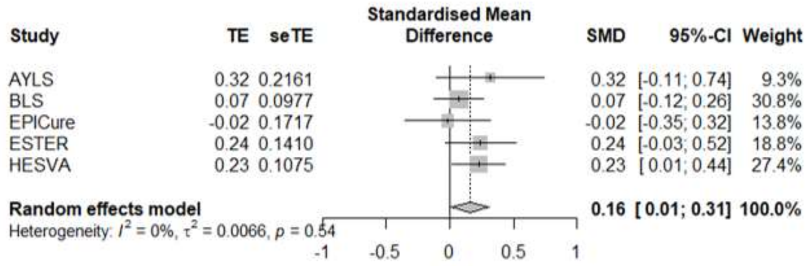

Conscientiousness

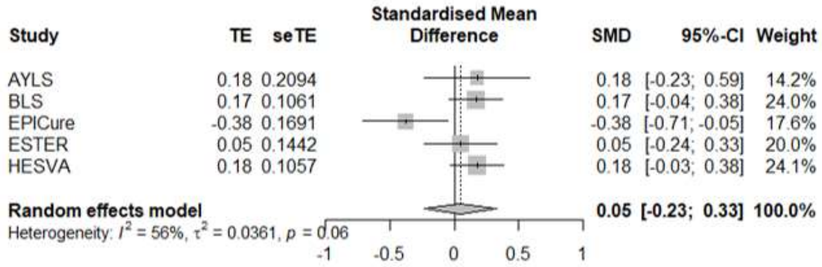

Fig. S1. Sensitivity analysis: Forest plots showing personality differences between VP/VLBW and term-born participants using only cohorts included in one-stage analysis.

Note. SMD = standardised mean difference; CI = confidence interval. AYLS = Arvo Ylppö Longitudinal Study; BLS = Bavarian Longitudinal Study; EPICure = EPICure Study; ESTER = ESTER preterm birth study; HeSVA = Helsinki Study of Very Low Birth Weight Adults.
