# Supplementary material for: The effect of very preterm birth on the Five-Factor Model of personality traits: A meta-analysis of individual participant data
Source: Eur J Pers. 2024 Oct 24;39(4):498–517. doi: 10.1177/08902070241280101 (PMC13029488; doi:10.1177/08902070241280101)
Supplement: Supplemental Material - The effect of very preterm birth on the five-factor model of personality traits: A meta-analysis of individual participant data [file sj-pdf-2-erp-10.1177_08902070241280101.pdf]

The effect of very preterm birth on the Five-Factor Model of personality traits: A meta-analysis of individual participant data

R script

#Load libraries

```
library(plyr)
library(haven)
library(tableone)
library(meta)
library(metafor)
library(lme4)
library(lmerTest)
library(Hmisc)
library(mice)
library(sjPlot)
library(mitml)
```

#Load files in (personality data for each cohort)

```
AYLS <- read_sav("Z:/M-Drive/PS/DWTeam/Eva Backup/Data/IPD Personality/AYLS_personality_zscore_bpd_bw_ga_age.sav")
BLS <- read_sav("Z:/H-Drive/Desktop/PhD/Data/IPD/Personality/Harmonisation/BLS_personality_zscore_bpd_bw_ga_age.sav")
EPICure <- read_sav("Z:/H-Drive/Desktop/PhD/Data/IPD/Personality/Harmonisation/EPICure_personality_zscore_controlvars_bpd_bw_ga_age.sav")
ESTER <- read_sav("Z:/H-Drive/Desktop/PhD/Data/IPD/Personality/Harmonisation/ESTER_personality_zscore_bpd_bw_ga_age.sav")
HESVA <- read_sav("Z:/H-Drive/Desktop/PhD/Data/IPD/Personality/Harmonisation/HESVA_personality_zscore_bpd_bw_ga_age.sav")
```

#Sample description: in VP/VLBW

```
"Chi-squared analysis: Stratify by VP/VLBW"
listVars <- c("sexn", "education", "assess_age_yrs_adulthood","ga_weeks", "birth_weight", "delivery_mode", "Fenton_Birthweight_Z", "Fenton_SGA_Group", "NSI", "bpd")
catVar = c("sexn", "education", "assess_age_yrs_adulthood","delivery_mode", "Fenton_SGA_Group", "NSI", "bpd")
table1 <- CreateTableOne(listVars, AYLS, catVar, strata = c("group_birth"))
table1
```

#Prepare for one stage IPD

"Specify cohort level information for each study (average age of assessment, year of cohort, drop out rate)"

```
AYLS$Age = 25.46
BLS$Age = 26.15
EPICure$Age = 19.25
ESTER$Age = 23.46
HESVA$Age = 24.56
```

AYLS$Year = 1985
BLS$Year = 1985.5
EPICure$Year = 1995
ESTER$Year = 1986
HESVA$Year = 1981.5
AYLS$dropout = 55.73
BLS$dropout = 61.53
EPICure$dropout = 65.83
ESTER$dropout = 8.31
HESVA$dropout = 42.78
"Bind datasets together"
IPD = rbind(AYLS, BLS, EPICure, ESTER, HESVA)

"Specify factors in the dataset"
IPD$Year = as.factor(IPD$Year)
IPD$Age = as.factor(IPD$Age)
IPD$dropout = as.factor(IPD$dropout)
IPD$group_birth = as.factor(IPD$group_birth)
IPD$delivery_mode = as.factor(IPD$delivery_mode)
IPD$sexn = as.factor(IPD$sexn)
IPD$education = as.factor(IPD$education)
IPD$NSI = as.factor(IPD$NSI)

#Multiple imputation
"Remove variables not necessary to the analysis (remove "Fenton_SGA_Group" as already have the continuous variable "Fenton_Birthweight_Z" which will be used*)"*
IPD_analysis = subset(IPD, select= -c(Fenton_SGA_Group))
"Specify imputation model"
init = mice(IPD_analysis, maxit=0)
meth = init$method
predM = init$predictorMatrix
predM[, c("subjido", "Age", "Year", "dropout")]=0
meth[c("subjido", "Age", "Year", "dropout", "pers_ope_mean_z_adult", "ga_weeks", "birth_weight")]=""
meth
set.seed(103)
IPD_imp = mice(IPD_analysis, method=meth, predictorMatrix = predM, m=40)

#One stage analysis (unadjusted): linear mixed model with fixed effect of VP and random effect of cohort (not using imputed dataset here as no data is missing when not adjusting for any covariates)

"Extraversion"
Extraversion = lmer(pers_ext_mean_z_adult ~group_birth + (1|Age) + (1|Year) + (1|dropout), data = IPD)
summary(Extraversion)

confint(Extraversion)
tab_model(Extraversion)

"Neuroticism"
Neuroticism = lmer(pers_neu_mean_z_adult ~ group_birth + (1|Age) + (1|Year) + (1|dropout), data = IPD)
summary(Neuroticism)
tab_model(Neuroticism)
confint(Neuroticism)

"Agreeableness"
Agreeableness = lmer(pers_agr_mean_z_adult ~ group_birth + (1|Age) + (1|Year) + (1|dropout), data = IPD)
summary(Agreeableness)
confint(Agreeableness)
tab_model(Agreeableness)

"Openness"
Openness = lmer(pers_ope_mean_z_adult ~ group_birth + (1|Age) + (1|Year) + (1|dropout), data = IPD)
summary(Openness)
confint(Openness)
tab_model(Openness)

"Conscientiousness"
Conscientiousness = lmer(pers_con_mean_z_adult ~ group_birth + (1|Age) + (1|Year) + (1|dropout), data = IPD)
summary(Conscientiousness)
confint(Conscientiousness)
tab_model(Conscientiousness)

#One stage analysis: Adjusted for sex and education (using imputed dataset)

Extraversion_imp = with(IPD_imp, lme4::lmer(pers_ext_mean_z_adult ~group_birth + sexn + education + (1|Age) + (1|Year) + (1|dropout)))
summary(pool(Extraversion_imp))

Neuroticism_imp = with(IPD_imp, lme4::lmer(pers_neu_mean_z_adult ~group_birth + sexn + education + (1|Age) + (1|Year) + (1|dropout)))
summary(pool(Neuroticism_imp))

Agreeableness_imp = with(IPD_imp, lme4::lmer(pers_agr_mean_z_adult ~group_birth + sexn + education + (1|Age) + (1|Year) + (1|dropout)))
summary(pool(Agreeableness_imp))

Openness_imp = with(IPD_imp, lme4::lmer(pers_ope_mean_z_adult ~group_birth + sexn + education + (1|Age) + (1|Year) + (1|dropout)))
summary(pool(Openness_imp))

Conscientiousness_imp = with(IPD_imp, lme4::lmer(pers_con_mean_z_adult ~group_birth + sexn + education + (1|Age) + (1|Year) + (1|dropout)))

summary(pool(Conscientiousness_imp))

#One stage analysis: Interaction with sex and education

Extraversion_imp = with(IPD_imp, lme4::lmer(pers_ext_mean_z_adult ~group_birth*sexn + group_birth*education + (1|Age) + (1|Year) + (1|dropout)))
summary(pool(Extraversion_imp))

Neuroticism_imp = with(IPD_imp, lme4::lmer(pers_neu_mean_z_adult ~group_birth*sexn + group_birth*education + (1|Age) + (1|Year) + (1|dropout)))
summary(pool(Neuroticism_imp))

Agreeableness_imp = with(IPD_imp, lme4::lmer(pers_agr_mean_z_adult ~group_birth*sexn + group_birth*education + (1|Age) + (1|Year) + (1|dropout)))
summary(pool(Agreeableness_imp))

Openness_imp = with(IPD_imp, lme4::lmer(pers_ope_mean_z_adult ~group_birth*sexn + group_birth*education + (1|Age) + (1|Year) + (1|dropout)))
summary(pool(Openness_imp))

Conscientiousness_imp = with(IPD_imp, lme4::lmer(pers_con_mean_z_adult ~group_birth*sexn + group_birth*education + (1|Age) + (1|Year) + (1|dropout)))
summary(pool(Conscientiousness_imp))

#Subgroup analyses: Remove those with NSI

library(dplyr)
IPD_noNSI = IPD_analysis %>%
filter(NSI != 1)
IPD_noNSI = subset(IPD_noNSI, select=-c(NSI))

"Repeat above one stage analyses"

Extraversion = lmer(pers_ext_mean_z_adult ~group_birth + (1|Age) + (1|Year) + (1|dropout), data = IPD_noNSI)
summary(Extraversion)
confint(Extraversion)
tab_model(Extraversion)

Neuroticism = lmer(pers_neu_mean_z_adult ~ group_birth + (1|Age) + (1|Year) + (1|dropout), data = IPD_noNSI)
summary(Neuroticism)
tab_model(Neuroticism)
confint(Neuroticism)

Agreeableness = lmer(pers_agr_mean_z_adult ~ group_birth + (1|Age) + (1|Year) + (1|dropout), data = IPD_noNSI)
summary(Agreeableness)
confint(Agreeableness)
tab_model(Agreeableness)

```
Openness = lmer(pers_ope_mean_z_adult ~ group_birth + (1|Age) + (1|Year) + (1|dropout), data =
IPD_noNSI)
summary(Openness)
confint(Openness)
tab_model(Openness)

Conscientiousness = lmer(pers_con_mean_z_adult ~ group_birth + (1|Age) + (1|Year) + (1|dropout),
data = IPD_noNSI)
summary(Conscientiousness)
confint(Conscientiousness)
tab_model(Conscientiousness)
```

#Subgroup analysis: within VP/VLBW group only

"Select only VP/VLBW"

```
AYLS_VPVLBW = AYLS[AYLS$group_birth == 1, ]
BLS_VPVLBW = BLS[BLS$group_birth == 1, ]
EPICure_VPVLBW = EPICure[EPICure$group_birth == 1, ]
ESTER_VPVLBW = ESTER[ESTER$group_birth == 1, ]
HESVA_VPVLBW = HESVA[HESVA$group_birth == 1, ]
```

"Combine datasets together"

```
IPD_VPVLBW = rbind(AYLS_VPVLBW, BLS_VPVLBW, EPICure_VPVLBW, ESTER_VPVLBW,
HESVA_VPVLBW)
IPD_VPVLBW$Age = as.factor(IPD$Age)
IPD_VPVLBW$Year = as.factor(IPD$Year)
IPD_VPVLBW$dropout = as.factor(IPD$dropout)
```

"Correlation matrix between predictors"

```
IPD_cor = subset(IPD_VPVLBW, select=c(sexn:NSI))
mydata.cor = cor(IPD_cor, method = c("spearman"))
mydata.rcorr = rcorr(as.matrix(IPD_cor))
mydata.rcorr
mydata.coeff = mydata.rcorr$r
mydata.p = mydata.rcorr$P
mydata.coeff
mydata.p
```

#Repeat one stage analysis

"Remove variables not included in analysis"

```
IPD_VPVLBW_analysis = subset(IPD_VPVLBW, select= -c(Fenton_SGA_Group, NSI))
```

"Multiple imputation"

```
init = mice(IPD_VPVLBW_analysis, maxit=0)
meth = init$method
predM = init$predictorMatrix
predM[, c("subjido", "Age", "Year", "dropout")]=0
meth[c("subjido", "Age", "Year", "dropout")]=""
meth
```

```
set.seed(103)
IPD_VPVLBW_imp = mice(IPD_VPVLBW_analysis, method=meth, predictorMatrix = predM, m=40)
```

"Extraversion"

```
Extraversion_imp = with(IPD_VPVLBW_imp, lme4::lmer(pers_ext_mean_z_adult ~ ga_weeks + sexn
+ Fenton_Birthweight_Z + delivery_mode + education + bpd + (1|Age) + (1|Year) + (1|dropout)))
summary(pool(Extraversion_imp))
mod1=pool(Extraversion_imp)
pool1 = summary(pool(Extraversion_imp))
pool1$estimate - (1.96*pool1$std.error)
pool1$estimate + (1.96*pool1$std.error)
est = testEstimates(as.mitml.result(Extraversion_imp), extra.pars = T)$extra.pars
est
```

"Neuroticism"

```
Neuroticism_imp = with(IPD_VPVLBW_imp, lme4::lmer(pers_neu_mean_z_adult ~ ga_weeks + sexn
+ Fenton_Birthweight_Z + delivery_mode + education + bpd + (1|Age) + (1|Year) + (1|dropout)))
summary(pool(Neuroticism_imp))
pool2 = summary(pool(Neuroticism_imp))
pool2$estimate - (1.96*pool2$std.error)
pool2$estimate + (1.96*pool2$std.error)
est = testEstimates(as.mitml.result(Neuroticism_imp), extra.pars = T)$extra.pars
est
```

"Agreeableness"

```
Agreeableness_imp = with(IPD_VPVLBW_imp, lme4::lmer(pers_agr_mean_z_adult ~ ga_weeks +
sexn + Fenton_Birthweight_Z + delivery_mode + education + bpd + (1|Age) + (1|Year) +
(1|dropout)))
summary(pool(Agreeableness_imp))
pool3 = summary(pool(Agreeableness_imp))
pool3$estimate - (1.96*pool3$std.error)
pool3$estimate + (1.96*pool3$std.error)
est = testEstimates(as.mitml.result(Agreeableness_imp), extra.pars = T)$extra.pars
est
```

"Openness"

```
Openness_imp = with(IPD_VPVLBW_imp, lme4::lmer(pers_ope_mean_z_adult ~ ga_weeks + sexn +
Fenton_Birthweight_Z + delivery_mode + education + bpd + (1|Age) + (1|Year) + (1|dropout)))
summary(pool(Openness_imp))
pool4 = summary(pool(Openness_imp))
pool4$estimate - (1.96*pool4$std.error)
pool4$estimate + (1.96*pool4$std.error)
est = testEstimates(as.mitml.result(Openness_imp), extra.pars = T)$extra.pars
est
```

"Conscientiousness"

```
Conscientiousness_imp = with(IPD_VPVLBW_imp, lme4::lmer(pers_con_mean_z_adult ~ ga_weeks +
sexn + Fenton_Birthweight_Z + delivery_mode + education + bpd + (1|Age) + (1|Year) +
(1|dropout)))
summary(pool(Conscientiousness_imp))
pool5 = summary(pool(Conscientiousness_imp))
```

pool5$estimate - (1.96*pool5$std.error)
pool5$estimate + (1.96*pool5$std.error)
est = testEstimates(as.mitml.result(Conscientiousness_imp), extra.pars = T)$extra.pars
est

#Subgroup analysis: within VP/VLBW group only and removing NSI
IPD_VPVLBW_noNSI_analysis = subset(IPD_VPVLBW, select= -c(Fenton_SGA_Group))
library(dplyr)
IPD_VPVLBW_noNSI = IPD_VPVLBW_noNSI_analysis %>%
filter(NSI != 1)
IPD_VPVLBW_noNSI = subset(IPD_VPVLBW_noNSI, select= -c(NSI))

"Multiple imputation"
init = mice(IPD_VPVLBW_noNSI, maxit=0)
meth = init$method
predM = init$predictorMatrix
predM[, c("subjido", "Age", "Year", "dropout")]=0
meth[c("subjido", "Age", "Year", "dropout")]=""
meth
set.seed(103)
IPD_VPVLBW_noNSI_imp = mice(IPD_VPVLBW_noNSI, method=meth, predictorMatrix = predM, m=40)

"Extraversion"
Extraversion_imp = with(IPD_VPVLBW_noNSI_imp, lme4::lmer(pers_ext_mean_z_adult ~ ga_weeks + sexn + Fenton_Birthweight_Z + delivery_mode + education + bpd + (1|Age) + (1|Year) + (1|dropout)))
summary(pool(Extraversion_imp))
pool(Extraversion_imp)
pool1 = summary(pool(Extraversion_imp))
pool1$estimate - (1.96*pool1$std.error)
pool1$estimate + (1.96*pool1$std.error)
est = testEstimates(as.mitml.result(Extraversion_imp), extra.pars = T)$extra.pars
est

"Neuroticism"
Neuroticism_imp = with(IPD_VPVLBW_noNSI_imp, lme4::lmer(pers_neu_mean_z_adult ~ ga_weeks + sexn + Fenton_Birthweight_Z + delivery_mode + education + bpd + (1|Age) + (1|Year) + (1|dropout)))
summary(pool(Neuroticism_imp))
pool2 = summary(pool(Neuroticism_imp))
pool2$estimate - (1.96*pool2$std.error)
pool2$estimate + (1.96*pool2$std.error)
est = testEstimates(as.mitml.result(Neuroticism_imp), extra.pars = T)$extra.pars
est

"Agreeableness"
Agreeableness_imp = with(IPD_VPVLBW_noNSI_imp, lme4::lmer(pers_agr_mean_z_adult ~ ga_weeks + sexn + Fenton_Birthweight_Z + delivery_mode + education + bpd + (1|Age) + (1|Year) + (1|dropout)))
summary(pool(Agreeableness_imp))

pool3 = summary(pool(Agreeableness_imp))
pool3$estimate - (1.96*pool3$std.error)
pool3$estimate + (1.96*pool3$std.error)
est = testEstimates(as.mitml.result(Agreeableness_imp), extra.pars = T)$extra.pars
est

"Openness"
Openness_imp = with(IPD_VPVLBW_noNSI_imp, lme4::lmer(pers_ope_mean_z_adult ~ ga_weeks + sexn + Fenton_Birthweight_Z + delivery_mode + education + bpd + (1|Age) + (1|Year) + (1|dropout)))
summary(pool(Openness_imp))
pool4 = summary(pool(Openness_imp))
pool4$estimate - (1.96*pool4$std.error)
pool4$estimate + (1.96*pool4$std.error)
est = testEstimates(as.mitml.result(Openness_imp), extra.pars = T)$extra.pars
est

"Conscientiousness"
Conscientiousness_imp = with(IPD_VPVLBW_noNSI_imp, lme4::lmer(pers_con_mean_z_adult ~ ga_weeks + sexn + Fenton_Birthweight_Z + delivery_mode + education + bpd + (1|Age) + (1|Year) + (1|dropout)))
summary(pool(Conscientiousness_imp))
pool5 = summary(pool(Conscientiousness_imp))
pool5$estimate - (1.96*pool5$std.error)
pool5$estimate + (1.96*pool5$std.error)
est = testEstimates(as.mitml.result(Conscientiousness_imp), extra.pars = T)$extra.pars
est

#Sensitivity analyses: BPD defined as >28 days oxygen supply after birth

"Only EPICure had a different definition, so repeat above analyses by removing EPICure"
IPD_VPVLBW = rbind(AYLS_VPVLBW, BLS_VPVLBW, ESTER_VPVLBW, HESVA_VPVLBW)
IPD_VPVLBW_analysis = subset(IPD_VPVLBW, select= -c(Fenton_SGA_Group, NSI))

"Multiple imputation"
init = mice(IPD_VPVLBW_analysis, maxit=0)
meth = init$method
predM = init$predictorMatrix
predM[, c("subjido", "Age", "Year", "dropout")]=0
meth[c("subjido", "Age", "Year", "dropout")]=""
meth
set.seed(103)
IPD_VPVLBW_imp = mice(IPD_VPVLBW_analysis, method=meth, predictorMatrix = predM, m=40)

"Extraversion"
Extraversion_imp = with(IPD_VPVLBW_imp, lme4::lmer(pers_ext_mean_z_adult ~ ga_weeks + sexn + Fenton_Birthweight_Z + delivery_mode + education + bpd + (1|Age) + (1|Year) + (1|dropout)))
summary(pool(Extraversion_imp))
mod1=pool(Extraversion_imp)
pool1 = summary(pool(Extraversion_imp))
pool1$estimate - (1.96*pool1$std.error)

```
pool1$estimate + (1.96*pool1$std.error)
est = testEstimates(as.mitml.result(Extraversion_imp), extra.pars = T)$extra.pars
est
```

"Neuroticism"

```
Neuroticism_imp = with(IPD_VPVLBW_imp, lme4::lmer(pers_neu_mean_z_adult ~ ga_weeks + sexn
+ Fenton_Birthweight_Z + delivery_mode + education + bpd + (1|Age) + (1|Year) + (1|dropout)))
summary(pool(Neuroticism_imp))
pool2 = summary(pool(Neuroticism_imp))
pool2$estimate - (1.96*pool2$std.error)
pool2$estimate + (1.96*pool2$std.error)
est = testEstimates(as.mitml.result(Neuroticism_imp), extra.pars = T)$extra.pars
est
```

"Agreeableness"

```
Agreeableness_imp = with(IPD_VPVLBW_imp, lme4::lmer(pers_agr_mean_z_adult ~ ga_weeks +
sexn + Fenton_Birthweight_Z + delivery_mode + education + bpd + (1|Age) + (1|Year) +
(1|dropout)))
summary(pool(Agreeableness_imp))
pool3 = summary(pool(Agreeableness_imp))
pool3$estimate - (1.96*pool3$std.error)
pool3$estimate + (1.96*pool3$std.error)
est = testEstimates(as.mitml.result(Agreeableness_imp), extra.pars = T)$extra.pars
est
```

"Openness"

```
Openness_imp = with(IPD_VPVLBW_imp, lme4::lmer(pers_ope_mean_z_adult ~ ga_weeks + sexn +
Fenton_Birthweight_Z + delivery_mode + education + bpd + (1|Age) + (1|Year) + (1|dropout)))
summary(pool(Openness_imp))
pool4 = summary(pool(Openness_imp))
pool4$estimate - (1.96*pool4$std.error)
pool4$estimate + (1.96*pool4$std.error)
est = testEstimates(as.mitml.result(Openness_imp), extra.pars = T)$extra.pars
est
```

"Conscientiousness"

```
Conscientiousness_imp = with(IPD_VPVLBW_imp, lme4::lmer(pers_con_mean_z_adult ~ ga_weeks +
sexn + Fenton_Birthweight_Z + delivery_mode + education + bpd + (1|Age) + (1|Year) +
(1|dropout)))
summary(pool(Conscientiousness_imp))
pool5 = summary(pool(Conscientiousness_imp))
pool5$estimate - (1.96*pool5$std.error)
pool5$estimate + (1.96*pool5$std.error)
est = testEstimates(as.mitml.result(Conscientiousness_imp), extra.pars = T)$extra.pars
est
```

#Sensitivity analyses: Remove VPVLBW participants with NSI (within BPD defined as >28 days oxygen supply after birth subgroup)

```
IPD_VPVLBW_noNSI_analysis = subset(IPD_VPVLBW, select= -c(Fenton_SGA_Group))
```

```
library(dplyr)
IPD_VPVLBW_noNSI = IPD_VPVLBW_noNSI_analysis %>%
filter(NSI != 1)
IPD_VPVLBW_noNSI = subset(IPD_VPVLBW_noNSI, select= -c(NSI))
```

"Multiple imputation"

```
init = mice(IPD_VPVLBW_noNSI, maxit=0)
meth = init$method
predM = init$predictorMatrix
predM[, c("subjido", "Age", "Year", "dropout")]=0
meth[c("subjido", "Age", "Year", "dropout")]=""
meth
set.seed(103)
IPD_VPVLBW_noNSI_imp = mice(IPD_VPVLBW_noNSI, method=meth, predictorMatrix = predM,
m=40)
```

"Extraversion"

```
Extraversion_imp = with(IPD_VPVLBW_noNSI_imp, lme4::lmer(pers_ext_mean_z_adult ~ ga_weeks
+ sexn + Fenton_Birthweight_Z + delivery_mode + education + bpd + (1|Age) + (1|Year) +
(1|dropout)))
summary(pool(Extraversion_imp))
pool(Extraversion_imp)
pool1 = summary(pool(Extraversion_imp))
pool1$estimate - (1.96*pool1$std.error)
pool1$estimate + (1.96*pool1$std.error)
est = testEstimates(as.mitml.result(Extraversion_imp), extra.pars = T)$extra.pars
est
```

"Neuroticism"

```
Neuroticism_imp = with(IPD_VPVLBW_noNSI_imp, lme4::lmer(pers_neu_mean_z_adult ~ ga_weeks
+ sexn + Fenton_Birthweight_Z + delivery_mode + education + bpd + (1|Age) + (1|Year) +
(1|dropout)))
summary(pool(Neuroticism_imp))
pool2 = summary(pool(Neuroticism_imp))
pool2$estimate - (1.96*pool2$std.error)
pool2$estimate + (1.96*pool2$std.error)
est = testEstimates(as.mitml.result(Neuroticism_imp), extra.pars = T)$extra.pars
est
```

"Agreeableness"

```
Agreeableness_imp = with(IPD_VPVLBW_noNSI_imp, lme4::lmer(pers_agr_mean_z_adult ~
ga_weeks + sexn + Fenton_Birthweight_Z + delivery_mode + education + bpd + (1|Age) + (1|Year) +
(1|dropout)))
summary(pool(Agreeableness_imp))
pool3 = summary(pool(Agreeableness_imp))
pool3$estimate - (1.96*pool3$std.error)
pool3$estimate + (1.96*pool3$std.error)
est = testEstimates(as.mitml.result(Agreeableness_imp), extra.pars = T)$extra.pars
est
```

"Openness"

```
Openness_imp = with(IPD_VPVLBW_noNSI_imp, lme4::lmer(pers_ope_mean_z_adult ~ ga_weeks + sexn + Fenton_Birthweight_Z + delivery_mode + education + bpd + (1|Age) + (1|Year) + (1|dropout)))
summary(pool(Openness_imp))
pool4 = summary(pool(Openness_imp))
pool4$estimate - (1.96*pool4$std.error)
pool4$estimate + (1.96*pool4$std.error)
est = testEstimates(as.mitml.result(Openness_imp), extra.pars = T)$extra.pars
est
```

"Conscientiousness"

```
Conscientiousness_imp = with(IPD_VPVLBW_noNSI_imp, lme4::lmer(pers_con_mean_z_adult ~ ga_weeks + sexn + Fenton_Birthweight_Z + delivery_mode + education + bpd + (1|Age) + (1|Year) + (1|dropout)))
summary(pool(Conscientiousness_imp))
pool5 = summary(pool(Conscientiousness_imp))
pool5$estimate - (1.96*pool5$std.error)
pool5$estimate + (1.96*pool5$std.error)
est = testEstimates(as.mitml.result(Conscientiousness_imp), extra.pars = T)$extra.pars
est
```

#Sensitivity analysis: Two stage analysis including estimates from Danish cohort (save estimates from each cohort)

"Extraversion"

```
AYLS_Ext = t.test(pers_ext_mean_z_adult ~ group_birth, AYLS)
AYLS_Ext_md = AYLS_Ext$estimate[2]
AYLS_Ext_se = AYLS_Ext$stderr
BLS_Ext = t.test(pers_ext_mean_z_adult ~ group_birth, BLS)
BLS_Ext_md = BLS_Ext$estimate[2]
BLS_Ext_se = BLS_Ext$stderr
EPICure_Ext = t.test(pers_ext_mean_z_adult ~ group_birth, EPICure)
EPICure_Ext_md = EPICure_Ext$estimate[2]
EPICure_Ext_se = EPICure_Ext$stderr
ESTER_Ext = t.test(pers_ext_mean_z_adult ~ group_birth, ESTER)
ESTER_Ext_md = ESTER_Ext$estimate[2]
ESTER_Ext_se = ESTER_Ext$stderr
HESVA_Ext = t.test(pers_ext_mean_z_adult ~ group_birth, HESVA)
HESVA_Ext_md = HESVA_Ext$estimate[2]
HESVA_Ext_se = HESVA_Ext$stderr
Den_Ext_md = -0.2964
Den_Ext_se= 0.0969
```

"Group all estimates together into one dataset"

```
Cohort1 = c("AYLS", "BLS", "EPICure", "ESTER", "HESVA")
TE = c(AYLS_Ext_md, BLS_Ext_md, EPICure_Ext_md, ESTER_Ext_md, HESVA_Ext_md)
seTE = c(AYLS_Ext_se, BLS_Ext_se, EPICure_Ext_se, ESTER_Ext_se, HESVA_Ext_se)
Country = c("Finland", "Germany", "UK", "Finland", "Finland")
Extraversion = cbind(Cohort1, TE, seTE, Country)
rownames(Extraversion) = NULL
```

```
Extraversion = as.data.frame(Extraversion)
Extraversion$Cohort1 = c("AYLS", "BLS", "EPICure", "ESTER", "HESVA")
toString(Extraversion$Cohort1, width = 8)
Extraversion$TE = as.character(Extraversion$TE)
Extraversion$TE = as.numeric(Extraversion$TE)
Extraversion$seTE = as.character(Extraversion$seTE)
Extraversion$seTE = as.numeric(Extraversion$seTE)
Extraversion$Country = as.character(Extraversion$Country)
```

"Two-stage IPD with forest plot"

```
Extraversion_meta = metagen(TE, seTE, data = Extraversion, studlab = paste(Cohort1), comb.fixed =
FALSE, comb.random = TRUE, method.tau = "SJ", hakn = TRUE, prediction = FALSE, sm = "SMD")
Extraversion_meta
forest(Extraversion_meta, xlim = c(-1, 1))
```

"Neuroticism"

```
AYLS_Neu = t.test(pers_neu_mean_z_adult ~ group_birth, AYLS)
AYLS_Neu_md = AYLS_Neu$estimate[2]
AYLS_Neu_se = AYLS_Neu$stderr
BLS_Neu = t.test(pers_neu_mean_z_adult ~ group_birth, BLS)
BLS_Neu_md = BLS_Neu$estimate[2]
BLS_Neu_se = BLS_Neu$stderr
EPICure_Neu = t.test(pers_neu_mean_z_adult ~ group_birth, EPICure)
EPICure_Neu_md = EPICure_Neu$estimate[2]
EPICure_Neu_se = EPICure_Neu$stderr
ESTER_Neu = t.test(pers_neu_mean_z_adult ~ group_birth, ESTER)
ESTER_Neu_md = ESTER_Neu$estimate[2]
ESTER_Neu_se = ESTER_Neu$stderr
HESVA_Neu = t.test(pers_neu_mean_z_adult ~ group_birth, HESVA)
HESVA_Neu_md = HESVA_Neu$estimate[2]
HESVA_Neu_se = HESVA_Neu$stderr
Den_Neu_md = 0.2681
Den_Neu_se= 0.0968
```

"Group all estimates into one dataset"

```
Cohort1 = c("AYLS", "BLS", "EPICure", "ESTER", "HESVA")
TE = c(AYLS_Neu_md, BLS_Neu_md, EPICure_Neu_md, ESTER_Neu_md, HESVA_Neu_md)
seTE = c(AYLS_Neu_se, BLS_Neu_se, EPICure_Neu_se, ESTER_Neu_se, HESVA_Neu_se)
Country = c("Finland", "Germany", "UK", "Finland", "Finland")
Neuroticism = cbind(Cohort1, TE, seTE, Country)
rownames(Neuroticism) = NULL
Neuroticism = as.data.frame(Neuroticism)
Neuroticism$Cohort1 = c("AYLS", "BLS", "EPICure", "ESTER", "HESVA")
toString(Neuroticism$Cohort1, width = 8)
Neuroticism$TE = as.character(Neuroticism$TE)
Neuroticism$TE = as.numeric(Neuroticism$TE)
Neuroticism$seTE = as.character(Neuroticism$seTE)
Neuroticism$seTE = as.numeric(Neuroticism$seTE)
Neuroticism$Country = as.character(Neuroticism$Country)
```

"Two stage IPD with forest plot"

```
Neuroticism_meta = metagen(TE, seTE, data = Neuroticism, studlab = paste(Cohort1), comb.fixed =
FALSE, comb.random = TRUE, method.tau = "SJ", hakn = TRUE, prediction = FALSE, sm = "SMD")
Neuroticism_meta
forest(Neuroticism_meta, xlim = c(-1, 1))

"Agreeableness"
AYLS_Agr = t.test(pers_agr_mean_z_adult ~ group_birth, AYLS)
AYLS_Agr_md = AYLS_Agr$estimate[2]
AYLS_Agr_se = AYLS_Agr$stderr
BLS_Agr = t.test(pers_agr_mean_z_adult ~ group_birth, BLS)
BLS_Agr_md = BLS_Agr$estimate[2]
BLS_Agr_se = BLS_Agr$stderr
EPICure_Agr = t.test(pers_agr_mean_z_adult ~ group_birth, EPICure)
EPICure_Agr_md = EPICure_Agr$estimate[2]
EPICure_Agr_se = EPICure_Agr$stderr
ESTER_Agr = t.test(pers_agr_mean_z_adult ~ group_birth, ESTER)
ESTER_Agr_md = ESTER_Agr$estimate[2]
ESTER_Agr_se = ESTER_Agr$stderr
HESVA_Agr = t.test(pers_agr_mean_z_adult ~ group_birth, HESVA)
HESVA_Agr_md = HESVA_Agr$estimate[2]
HESVA_Agr_se = HESVA_Agr$stderr
Den_Agr_md = 0.2503
Den_Agr_se= 0.0967

“Group all estimates into one dataset”
Cohort1 = c("AYLS", "BLS", "EPICure", "ESTER", "HESVA")
TE = c(AYLS_Agr_md, BLS_Agr_md, EPICure_Agr_md, ESTER_Agr_md, HESVA_Agr_md)
seTE = c(AYLS_Agr_se, BLS_Agr_se, EPICure_Agr_se, ESTER_Agr_se, HESVA_Agr_se)
Country = c("Finland", "Germany", "UK", "Finland", "Finland")
Agreeableness = cbind(Cohort1, TE, seTE, Country)
rownames(Agreeableness) = NULL
Agreeableness = as.data.frame(Agreeableness)
Agreeableness$Cohort1 = c("AYLS", "BLS", "EPICure", "ESTER", "HESVA")
toString(Agreeableness$Cohort1, width = 8)
Agreeableness$TE = as.character(Agreeableness$TE)
Agreeableness$TE = as.numeric(Agreeableness$TE)
Agreeableness$seTE = as.character(Agreeableness$seTE)
Agreeableness$seTE = as.numeric(Agreeableness$seTE)
Agreeableness$Country = as.character(Agreeableness$Country)

“Two stage IPD with forest plot”
Agreeableness_meta = metagen(TE, seTE, data = Agreeableness, studlab = paste(Cohort1),
comb.fixed = FALSE, comb.random = TRUE, method.tau = "SJ", hakn = TRUE, prediction = FALSE, sm =
"SMD")
Agreeableness_meta
forest(Agreeableness_meta, xlim = c(-1, 1))

"Openness"
AYLS_Ope = t.test(pers_ope_mean_z_adult ~ group_birth, AYLS)
AYLS_Ope_md = AYLS_Ope$estimate[2]
AYLS_Ope_se = AYLS_Ope$stderr
```

```
BLS_Ope = t.test(pers_ope_mean_z_adult ~ group_birth, BLS)
BLS_Ope_md = BLS_Ope$estimate[2]
BLS_Ope_se = BLS_Ope$stderr
EPICure_Ope = t.test(pers_ope_mean_z_adult ~ group_birth, EPICure)
EPICure_Ope_md = EPICure_Ope$estimate[2]
EPICure_Ope_se = EPICure_Ope$stderr
ESTER_Ope = t.test(pers_ope_mean_z_adult ~ group_birth, ESTER)
ESTER_Ope_md = ESTER_Ope$estimate[2]
ESTER_Ope_se = ESTER_Ope$stderr
HESVA_Ope = t.test(pers_ope_mean_z_adult ~ group_birth, HESVA)
HESVA_Ope_md = HESVA_Ope$estimate[2]
HESVA_Ope_se = HESVA_Ope$stderr
Den_Ope_md = -0.1377
Den_Ope_se= 0.0965

"Group all estimates into one dataset"
Cohort1 = c("AYLS", "BLS", "EPICure", "ESTER", "HESVA")
TE = c(AYLS_Ope_md, BLS_Ope_md, EPICure_Ope_md, ESTER_Ope_md, HESVA_Ope_md)
seTE = c(AYLS_Ope_se, BLS_Ope_se, EPICure_Ope_se, ESTER_Ope_se, HESVA_Ope_se)
Country = c("Finland", "Germany", "UK", "Finland", "Finland")
Openness = cbind(Cohort1, TE, seTE, Country)
rownames(Openness) = NULL
Openness = as.data.frame(Openness)
Openness$Cohort1 = c("AYLS", "BLS", "EPICure", "ESTER", "HESVA")
toString(Openness$Cohort1, width = 8)
Openness$TE = as.character(Openness$TE)
Openness$TE = as.numeric(Openness$TE)
Openness$seTE = as.character(Openness$seTE)
Openness$seTE = as.numeric(Openness$seTE)
Openness$Country = as.character(Openness$Country)

"Two stage IPD with forest plot"
Openness_meta = metagen(TE, seTE, data = Openness, studlab = paste(Cohort1), comb.fixed =
FALSE, comb.random = TRUE, method.tau = "SJ", hakn = TRUE, prediction = FALSE, sm = "SMD")
Openness_meta
forest(Openness_meta, xlim = c(-1, 1))

"Conscientiousness"
AYLS_Con = t.test(pers_con_mean_z_adult ~ group_birth, AYLS)
AYLS_Con_md = AYLS_Con$estimate[2]
AYLS_Con_se = AYLS_Con$stderr
BLS_Con = t.test(pers_con_mean_z_adult ~ group_birth, BLS)
BLS_Con_md = BLS_Con$estimate[2]
BLS_Con_se = BLS_Con$stderr
EPICure_Con = t.test(pers_con_mean_z_adult ~ group_birth, EPICure)
EPICure_Con_md = EPICure_Con$estimate[2]
EPICure_Con_se = EPICure_Con$stderr
ESTER_Con = t.test(pers_con_mean_z_adult ~ group_birth, ESTER)
ESTER_Con_md = ESTER_Con$estimate[2]
ESTER_Con_se = ESTER_Con$stderr
HESVA_Con = t.test(pers_con_mean_z_adult ~ group_birth, HESVA)
```

```
HESVA_Con_md = HESVA_Con$estimate[2]
HESVA_Con_se = HESVA_Con$stderr
Den_Con_md = -0.0504
Den_Con_se= 0.0964
```

“Group all estimates into one dataset”

```
Cohort1 = c("AYLS", "BLS", "EPICure", "ESTER", "HESVA")
TE = c(AYLS_Con_md, BLS_Con_md, EPICure_Con_md, ESTER_Con_md, HESVA_Con_md)
seTE = c(AYLS_Con_se, BLS_Con_se, EPICure_Con_se, ESTER_Con_se, HESVA_Con_se)
Country = c("Finland", "Germany", "UK", "Finland", "Finland")
Conscientiousness = cbind(Cohort1, TE, seTE, Country)
rownames(Conscientiousness) = NULL
Conscientiousness = as.data.frame(Conscientiousness)
Conscientiousness$Cohort1 = c("AYLS", "BLS", "EPICure", "ESTER", "HESVA")
toString(Conscientiousness$Cohort1, width = 8)
Conscientiousness
Conscientiousness$TE = as.character(Conscientiousness$TE)
Conscientiousness$TE = as.numeric(Conscientiousness$TE)
Conscientiousness$seTE = as.character(Conscientiousness$seTE)
Conscientiousness$seTE = as.numeric(Conscientiousness$seTE)
Conscientiousness$Country = as.character(Conscientiousness$Country)
```

“Two stage IPD with forest plot”

```
Conscientiousness_meta = metagen(TE, seTE, data = Conscientiousness, studlab = paste(Cohort1), comb.fixed = FALSE, comb.random = TRUE, method.tau = "SJ", hakn = TRUE, prediction = FALSE, sm = "SMD")
Conscientiousness_meta
forest(Conscientiousness_meta, xlim = c(-1, 1))
```
